# LncRNA AL139294.1 can be transported by extracellular vesicles to promote the oncogenic behaviour of recipient cells through activation of the Wnt and NF-κB2 pathways in non-small-cell lung cancer

**DOI:** 10.1186/s13046-023-02939-z

**Published:** 2024-01-16

**Authors:** Xinyi Ma, Zhenhua Chen, Wei Chen, Ziyuan Chen, Yue Shang, Yikai Zhao, Leyi Li, Chengwei Zhou, Jinxian He, Xiaodan Meng

**Affiliations:** 1 Department of Biochemistry and Molecular Biology, School of Basic Medical Sciences, Health Science Center, Ningbo University, 818 Fenghua Road, Ningbo, Zhejiang 315211 China; 2 Zhejiang Provincial Key Laboratory of Pathophysiology, Health Science Center, Ningbo University, Ningbo, Zhejiang 315211 China; 3 Department of Thoracic Surgery, The First Affiliated Hospital of Ningbo University, Ningbo, Zhejiang 315020 China; 4 Department of Thoracic Surgery, The Ningbo Medical Center Lihuili Hospital, Ningbo University, Ningbo, 315048 China

**Keywords:** Extracellular vesicles, Long non-coding RNA, Non-small-cell lung cancer, Tumour progression, Tumour marker

## Abstract

**Background:**

Extracellular vesicles (EVs) participate in cancer development via cell-to-cell communication. Long non-coding RNAs (lncRNAs), one component of EVs, can play an essential role in non-small-cell lung cancer (NSCLC) through EV-mediated delivery.

**Methods:**

The NSCLC-associated lncRNA AL139294.1 in EVs was identified via lncRNA microarray analysis. The role of AL139294.1 in NSCLC was examined in vitro and in vivo. Confocal microscopy was used to observe the encapsulation of AL139294.1 into EVs and its transport to recipient cells. A co-culture device was used to examine the effects of transported AL139294.1 on the oncogenic behaviour of recipient cells. Dual-luciferase reporter assay was performed to verify the direct interaction of miR-204-5p with AL139294.1 and bromodomain-containing protein 4 (BRD4). AL139294.1 and miR-204-5p in EVs were quantified using quantitative polymerase chain reaction. Receiver operating characteristic analyses were conducted to evaluate the diagnostic efficiency.

**Results:**

The lncRNA AL139294.1 in EVs promoted NSCLC progression in vitro and in vivo. After AL139294.1 was encapsulated into EVs and transported to recipient cells, it promoted the cells’ proliferation, migration, and invasion abilities by competitively binding with miR-204-5p to regulate BRD4, leading to the activation of the Wnt and NF-κB2 pathways. Additionally, the expression of serum lncRNA AL139294.1 in EVs was increased, whereas miR-204-5p in EVs was decreased in NSCLC. High levels of lncRNA AL139294.1 and low levels of miR-204-5p in EVs were associated with advanced pathological staging, lymph node metastasis, and distant metastasis, underscoring their promising utility for distinguishing between more and less severe manifestations of the disease.

**Conclusions:**

This study reveals a novel lncRNA in EVs associated with NSCLC, namely, AL139294.1, providing valuable insights into the development of NSCLC and introducing potential diagnostic biomarkers for NSCLC.

**Supplementary Information:**

The online version contains supplementary material available at 10.1186/s13046-023-02939-z.

## Background

Lung cancer has the highest mortality rate worldwide. Non-small-cell lung cancer (NSCLC) is the most common histological type of lung cancer, accounting for approximately 85% of total cases [1]. Lung adenocarcinoma (LUAD) and lung squamous cell carcinoma (LUSC) are the two main histological subtypes of NSCLC. Because most patients with lung cancer are at an advanced stage or have metastasis at diagnosis, the efficacy of existing treatment strategies, including surgery, chemotherapy, radiotherapy, immunotherapy, and targeted therapy, remains unsatisfactory. Moreover, the prognosis of lung cancer is poor, with the 5-year survival rate of patients being only 20–30%. Therefore, it is necessary to investigate the precise mechanisms underlying the development of lung cancer and identify novel targets for its diagnosis and treatment.

Extracellular vesicles (EVs) with a lipid bilayer membrane mediate cell-to-cell communication by transporting and releasing their contents, including microRNAs (miRNAs), long non-coding RNAs (lncRNAs), circular RNAs (circRNAs), messenger RNAs (mRNAs), DNAs and proteins, to recipient cells, thereby affecting the physiological and pathological functions of the cells [2, 3]. EVs are secreted by various cell types, and the specific proteins on their surface reflect their origin. Tumour cells are the predominant source of EVs [4]. At different stages of tumour development, EVs secreted by tumour cells participate in tumour growth, angiogenesis, epithelial–mesenchymal transition (EMT), metastasis, immune escape, and drug resistance via cell-to-cell communication [5, 6]. Therefore, in the past few decades, EVs have been intensively investigated to explore the mechanisms underlying tumorigenesis.

Abundant lncRNAs can be incorporated in EVs. At present, the exoRBase repository contains data on a total of 15,645 lncRNAs in EVs [7]. Studies have shown that lncRNAs regulate gene expression by interacting with DNAs, miRNAs, and proteins. Numerous dysregulated lncRNAs have been reported to participate in the occurrence and progression of cancer [8]. With regard to mechanism, lncRNAs frequently act as competitive endogenous RNAs (ceRNAs). They regulate the expression of cancer-related genes at the post-transcriptional level by competing with mRNAs for shared miRNAs, thereby blocking the inhibitory effects of miRNAs on their target mRNAs [9–11]. The ceRNA theory expands the gene regulatory network, with ceRNAs acting as oncogenes or tumour suppressors in various cancer types.

In the present study, we identified a novel NSCLC-associated lncRNA, namely, AL139294, which has not been reported in cancers or other diseases. AL139294.1 was found to be dramatically upregulated in the serum EVs of patients with NSCLC and could be transported by EVs to recipient cells. Transported AL139294.1 affected the proliferative, migratory, and invasive abilities of recipient cells by competitively binding with miR-204-5p to upregulate bromodomain-containing protein 4 (BRD4) and activate the Wnt and NF-κB2 pathways. Furthermore, we quantified AL139294.1 and miR-204-5p in serum samples EVs from healthy individuals, patients with pneumonia, and patients with NSCLC. The expression of AL139294.1 in EVs was negatively associated with that of miR-204-5p, and they showed diagnostic ability to discriminate patients with NSCLC from healthy individuals. Additionally, the high levels of AL139294.1 and the low levels of miR-204-5p in EVs were associated with the aggressive features of NSCLC. Altogether, this study reveals that the lncRNA AL139294.1 in EVs is a potential therapeutic and diagnostic target for lung cancer, particularly NSCLC.

## Methods

### Clinical specimens

Serum samples were randomly collected from patients with NSCLC (*n* = 111) and those with pneumonia (*n* = 49) before surgery or any treatment in the First Affiliated Hospital of Ningbo University (Ningbo, China) and Li Huili Hospital affiliated with Ningbo University (Ningbo, China) from 2019 to 2022. Pneumonia is a common lung disease. It was reported that EVs played vital roles in pneumonia [12, 13]. Therefore, we recruited the pneumonia samples to compare the levels of AL139294.1 and miR-204-5p in EVs among the NSCLC, healthy, and pneumonia subsets. In addition, a total of 40 serum samples were collected from healthy individuals with no history of any cancer in the Ningbo Kangning Hospital. Informed consent was obtained from each participant before sample collection. This study was approved by the Clinical Research Ethics Committee of the Health Science Center of Ningbo University and was performed in accordance with the principles of the Declaration of Helsinki. The clinical characteristics (age, sex, tumour size, histological subtype, TNM stage, lymph node metastasis status, and distant metastasis status) of all participants are presented in Table 1.

**Table 1:** The serum samples and the corresponding clinical parameters in the present study

Sample types and clinical data	Numbers n (%)
**Non-small Cell Lung Cancer**	**111 (%)**
Age (year)	
< 60	51 (45.9%)
≥ 60	60 (54.1%)
Gender	
Male	43 (38.7%)
Female	68 (61.3%)
Tumor size (cm)	
< 5	61 (55.0%)
≥ 5	18 (16.2%)
Unknown	32 (28.8%)
Histologic subtypes	
Squamous cell carcinoma	51 (45.9%)
Adenocarcinoma	60 (54.1%)
TNM stage	
I	8 (7.2%)
II	36 (32.4%)
III	44 (39.7%)
IV	23 (20.7%)
Distant metastasis	
M0	88 (79.3%)
M1	23 (20.7%)
Lymph node metastasis	
N0	38 (34.2%)
N1-3	73 (65.8%)
**Pneumonia**	**49 (100%)**
Age (year)	
< 60	16 (32.7%)
≥ 60	33 (67.3%)
Gender	
Male	22 (44.9%)
Female	27 (55.1%)
**Healthy**	**40 (100%)**
Age (year)	
< 60	17 (42.5%)
≥ 60	23 (57.5%)
Gender	
Male	22 (55.0%)
Female	18 (45.0%)

### Cell culture

All cell lines used in this study were obtained from the Cell Bank/Stem Cell Bank (Shanghai, China). The human normal lung epithelial cell line Beas-2B and the human embryonic kidney cell line HEK-293T were cultured in DMEM (Corning, United States of America). The human NSCLC cell lines LTEP-A2 and NCI-H1299 were cultured in RPMI-1640 medium (Corning, United States of America). The abovementioned media were supplemented with 10% foetal bovine serum (FBS; PAN, Germany), and 1% penicillin/streptomycin (New Cell & Molecular Biotech, China). The cells were cultured at 37 °C in a humidified incubator with 5% CO_2_. FBS was centrifuged (110,000 × g, 4 °C, 8 h) on an ultracentrifuge (BECKMAN COULTER, United States of America) to remove EVs.

### Vector construction and cell transfection

To investigate the role of AL139294.1 in lung cancer, AL139294.1-overexpression plasmids were constructed as described previously [14]. Briefly, the cDNA of AL139294.1 was subcloned into pcDNA3.1 vectors (Beijing Tsingke Biotech Co., Ltd., China) using specific primers to construct recombinant pcDNA3.1-AL139294.1 plasmids. The primer sequences are listed in Table S1. Finally, the recombinant AL139294.1-overexpression plasmids were verified through DNA sequencing (Beijing Genomics Institution, China). siRNAs targeting AL139294.1, miR-204-5p mimics, and miR-204-5p inhibitors were purchased from GenePharma (Shanghai, China). The plasmids, siRNAs, and miRNA mimics and inhibitors were transfected into cells using the Lipo8000™ transfection reagent (Beyotime, China) according to the manufacturer’s instructions. The sequences of negative control plasmids, negative control siRNAs, siRNAs, miRNA mimics, and inhibitors are listed in Table S1.

### LncRNA microarray analysis

LncRNAs in the serum EVs of healthy individuals (*n* = 3) and patients with NSCLC (*n* = 3) were profiled using the Arraystar Human LncRNA Array (Arraystar, Rockville, MD, United States of America) according to the Arraystar Super RNA Labeling protocol. Differentially expressed lncRNAs between healthy individuals and patients with NSCLC were identified based on the screening criteria of fold change values of > 2.0 and *P*-values of < 0.05.

### Cell proliferation assay

Cell counting kit-8 (CCK-8) (APExBIO, United States of America) was used to assess cell proliferation. Briefly, cells (2000 cells/well) were seeded in a 96-well plate (Jet Biofil, China) and transfected with plasmids, siRNAs, miRNA mimics, or miRNA inhibitors. At 24 h, 48 h, 72 h, and 96 h, 10 μL of CCK-8 reagent was added to each well, and the cells were incubated at 37 °C for 2 h. Subsequently, absorbance was measured at 450 nm on the iMark™ Microplate Reader (BIO-RAD, United States of America). The experiment was performed in triplicate.

### Wound healing assay

Beas-2B and NCI-H1299 cells were seeded in 6-well plates and transfected with plasmids, siRNAs, miRNA mimics, or miRNA inhibitors. When the cells reached approximately 95% confluence, a sterile pipette tip (200 μL) was used to create scratches on the cell monolayer. Subsequently, the cells were cultured in a medium containing 2% FBS. The movement of cells into the wound area was monitored and photographed at 0 h, 24 h, and 48 h using an optical microscope (Olympus, Japan). The distance of cell migration was measured using the Image-Pro Plus 6.0 software. This experiment was performed in triplicate.

### Transwell migration and invasion assays

Transwell migration and invasion assays were performed using transwell chambers (8-μm pore size; Corning, United States of America) coated without and with matrigel (BD Biosciences, United States of America), respectively. Transfected cells suspended in an FBS-free medium were added to the upper chamber, whereas 600 μL of a culture medium supplemented with 10% FBS was added to the lower chamber. After 24 h of incubation, the cells in the upper chamber were washed with phosphate-buffered saline (PBS), whereas the cells penetrating the chamber membrane were stained with 0.1% crystal violet (Solarbio, China). The stained cells were photographed using an optical microscope (Olympus, Japan) and counted using the ImageJ software (version 1.8.0, United States of America). Each experiment was performed in triplicate.

### Colony formation assay

Transfected cells (1000 cells/well) were cultured in a 6-well plate. The culture medium was replaced every 2 days. After 10–14 days of incubation, cell colonies in the 6-well plate were washed with PBS and stained with 0.1% crystal violet (Solarbio, China) for 30 min. The colonies were photographed using an optical microscope (Olympus, Japan) and counted using the ImageJ software (version 1.8.0, United States of America). This experiment was performed in triplicate.

### Cell apoptosis assay

Transfected cells were seeded in a 6-well plate and cultured for 24 h. EDTA-free trypsin was used to detach transfected cells from a 6-well plate. The cells were collected and resuspended in 500 μL of binding buffer. Subsequently, the cells were incubated with 5 μL of Annexin V-FITC and 10 μL of PI (MULTI SCIENCE, China) for 5 min at room temperature in the dark and examined on the CytoFlex S Flow Cytometer (BECKMAN, United States of America). This experiment was repeated at least three times. The apoptosis index refers to the ratio of the number of apoptotic cells and the number of all cells. Apoptotic cells include the early (Annexin V^+^) and the late (Annexin V^+^  + PI^+^) apoptotic cells.

### Cell cycle assay

For cell cycle assay, transfected cells were seeded in a 6-well plate at a density of 1 × 10^6^/well. The cells were collected, washed with PBS, and incubated with 1 mL of DNA staining solution PI (MULTI SCIENCE, China) and 10 μL of a permeabilisation solution for 30 min at room temperature in the dark. The stained cells were analysed on the CytoFlex S Flow Cytometer (BECKMAN, United States of America). The FlowJo (version 10.8.1) software was used for data analysis. This experiment was performed in triplicate.

### Western blotting

Total proteins were extracted from cells, tissues or EVs lysed with radioimmunoprecipitation assay (RIPA) buffer supplemented with 1-mM phenylmethylsulfonyl fluoride (PMSF) and phosphatase inhibitors (Beyotime, China) as described previously [15]. The concentration of extracted proteins was determined using a BCA protein assay kit (Beyotime, China) according to the manufacturer’s instructions. Subsequently, 30 μg of extracted proteins were loaded into each well of a 10% gel for separation. The separated proteins were transferred to a PVDF membrane (Millipore, United States of America), and the membrane was immersed in 5% non-fat milk for 1 h at room temperature to block non-specific binding. Thereafter, the membrane was incubated with primary antibodies overnight at 4 °C. The following day, the membrane was washed with TBST buffer and incubated with appropriate secondary antibodies for 1 h at room temperature. Finally, protein bands were visualised using an enhanced chemiluminescence (ECL) reagent (Advansta, United States of America) on an infrared imaging system (Li-COR, United States of America). The primary and secondary antibodies used for western blotting are listed in Table S2. This experiment was performed in triplicate.

### RNA extraction, cDNA synthesis, and quantitative polymerase chain reaction

Total RNA was isolated from cells, EVs, and tissues using TRIzol reagent (Invitrogen, USA) as described previously [16]. The extracted RNA was used to synthesize cDNA using the ReverTra Ace qPCR RT Master Mix with gDNA Remover (TOYOBO, Japan) according to the manufacturer’s instructions. For miRNAs, reverse transcription was performed using the miRNA First Strand cDNA Synthesis Kit (Vazyme Biotech Co., Ltd., China). A reaction mixture of 10 μL was prepared using 5 μL of Hieff® qPCR SYBR Green Master Mix (Yeasen Biotech, China), 3 μL of nuclease-free water, 0.5 μL of forward primer (10 μmol/L), 0.5 μL of reverse primer (10 μmol/L) and 1 μL of cDNA solution. Quantitative polymerase chain reaction (qPCR) was performed on the ABI 7500 real-time PCR system (Applied Biosystems, USA), with the reaction conditions set as follows: initial denaturation at 95 °C for 5 min, followed by 40 cycles of 94 °C for 30 s, 60 °C for 30 s and 72 °C for 30 s, then 98 °C for 10 min and a final hold at 4 °C. A melting curve was plotted and agarose gel electrophoresis was performed to examine the specificity of PCR products. Each reaction was run in triplicate. The primer sequences used for qPCR are shown in Table S3. GAPDH was used as an internal control for lncRNAs and mRNAs, whereas U6 was used as an internal control for miRNAs. For the relative quantification of nucleic acids in EVs, GAPDH is frequently chosen as the house-keeping gene to normalize the qPCR data [16, 17]. We also selected GAPDH as the house-keeping gene to normalize our lncRNA data in the present study. In the groups of healthy, pneumonia, and NSCLC, we calculated mean Cq values of 24.28 (SD = 0.53), 22.31 (SD = 1.77), and 23.80 (SD = 1.95), respectively. The relative expression of target RNAs was calculated using the 2^−ΔCq^ method, in which ΔCq was calculated by subtracting the mean Cq value of the corresponding internal control from the mean Cq value of the target RNA.

### Luciferase assay

The fragments of AL139294.1 (wild-type and mutant) or 3’-UTR of BRD4 (wild-type and mutant) were amplified and cloned into the pmirGLO dual-luciferase reporter gene vector (Promega, USA). The recombinant vectors were termed lncRNA AL139294.1-WT/MUT and BRD4-WT/MUT, respectively. HEK-293T cells were co-transfected with lncRNA AL139294.1-WT/MUT or BRD4-WT/MUT and miR-204-5p mimics or NC in a 12-well plate. After 48 h of incubation, a dual-luciferase reporter assay system (Promega, USA) was used to measure luciferase activity according to the manufacturer’s instructions. The Renilla luciferase activity was used as a control to normalise the firefly luciferase activity. This experiment was performed in triplicate.

### Isolation and characterisation of EVs

EVs secreted by cells were isolated from the culture medium (DMEM or RPMI-1640 containing 10% EV-depleted FBS) through differential ultracentrifugation as described previously [18]. Briefly, four sequential ultracentrifugation steps at 4 °C were included: 15 min at 300 × g to remove cells; 30 min at 10,000 × g to remove cell debris; ultracentrifugation at 110,000 × g for 70 min to precipitate EVs, followed by resuspension of EVs in PBS and their filtration with a membrane filter (0.22 μm in diameter); and final centrifugation at 110,000 × g for 70 min to remove soluble proteins. EVs were isolated from serum samples using the Total Exosome Isolation Reagent for Serum kit (Life Technologies, USA) according to the manufacturer’s instructions. The morphological characteristics and size of EVs were assessed using a scanning electron microscope (SU-70, HITACHI, Japan) and a potentiometric analyser (Zetasizer, ZEN3700, Malvern, England), respectively. Subsequently, western blotting was performed to test the presence of EV-specific proteins and EV-negative markers as described previously [17].

### Confocal microscopy

1,1'-dioctadecyl-3,3,3',3'-tetramethylindocarbocyanine perchlorate (Dil; Sigma, USA) was used to label EVs isolated from the conditioned medium. Dil and EV suspension were mixed in a ratio of 1:100, and the mixture was incubated for 30 min at room temperature. The mixture was centrifuged at 110,000 × g for 70 min at 4 °C to remove unbound Dil, and EVs were resuspended in PBS. Recipient cells were incubated with Dil-stained EVs for 12 h. Subsequently, the cells were washed twice with PBS, fixed with 4% paraformaldehyde (Solarbio, China) for 10 min at 4 °C, and stained with DAPI (Solarbio, China) for 5 min. The uptake of EVs by recipient cells was observed under a confocal microscope (LEICA TCS SP8, Germany).

### Bioinformatic analysis

To examine the expression pattern of BRD4 in NSCLC, we extracted a lung cancer dataset from The Cancer Genome Atlas (TCGA) database using the R software (version 4.2.1, USA). The Clinical Proteomic Tumor Analysis Consortium (CPTAC) database was used to analyse the protein expression of BRD4 in tumour and normal tissues and the correlation between BRD4 and lung cancer stages.

### Establishment of tumour xenograft models

A total of 14 female BALB/c nude mice (age, 6 weeks) were purchased from Beijing Vital River Laboratory Animal Technology Co., Ltd. To examine the influence of AL139294.1 in EVs on tumour growth, the mice were randomly divided into two groups, pcDNA3.1-EVs and lncRNA AL139294.1-EVs, with each group including 7 mice. A total of 1 × 10^7^ NCI-H1299 cells were injected under the axillary skin of nude mice. EVs were harvested from the culture medium of NCI-H1299 cells transfected with pcDNA3.1-lncRNA AL139294.1 plasmids or pcDNA3.1 empty vectors. After tumour formation, these EVs (5 μg, 100 μL) were injected into the mice via lateral tail veins every 7 days, 2 times. The mice were sacrificed 1 week after the last injection of EVs, and tumours were subsequently harvested. The maximum length (L), minimum length (W), and weight of tumours were measured, and tumour volume was calculated as ½LW^2^. All experiments involving animals were approved by the experimental animal ethics committee of the Health Science Center of Ningbo University.

### Statistical analysis

Statistical analysis was performed using the GraphPad Prism 9 and SPSS Statistics (version 22.0) software. For both in vitro and in vivo experiments, Student’s t-test was used to compare the data of the two groups. The data of the CCK-8 assay were analysed using two-way ANOVA. After the data were transformed to a normal distribution, ANOVA with Tukey’s HSD test was used to compare the levels of AL139294.1 and miR-204-5p in EVs among different groups (healthy, pneumonia, and NSCLC). In addition, Student’s t-test was used to compare the levels of AL139294.1 and miR-204-5p in EVs between two subgroups of NSCLC (smaller and bigger tumour sizes, LUSC and LUAD subtypes, lymph node metastasis-negative and -positive tumours, distant metastasis-negative and -positive tumours and early and advanced tumour stages). Receiver operating characteristic (ROC) curves were plotted and area under the curve (AUC) values were calculated to analyse the diagnostic value of AL139294.1 and miR-204-5p in EVs. Kaplan–Meier curves were plotted and the log-rank test was used to estimate overall survival, first-progression survival (the survival period from first diagnosis to first progression), and post-progression survival (the survival period from first progression to death). A two-tailed *P*-value of < 0.05 was considered statistically significant.

## Results

### Profiling of lncRNAs present in the serum EVs of patients with NSCLC

**Fig. 1:**
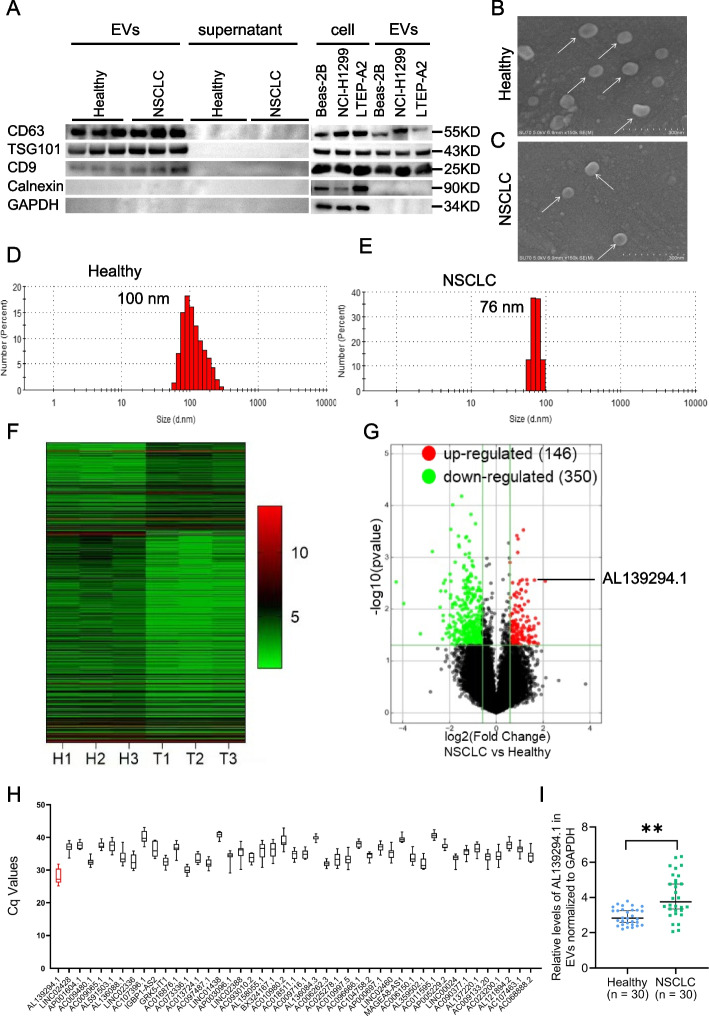
Screening of NSCLC-related lncRNAs in serum EVs. **A** Western blotting tested the specific markers of EVs (CD63, TSG101, and CD9) and EV-negative markers (Calnexin and GAPDH). Scanning electron microscopy was used to image the morphologies of serum EVs from healthy participants (**B**) and patients with NSCLC (**C**). A potentiometric analyzer was used to examine the size of serum EVs from healthy individuals (**D**) and patients with NSCLC (**E**). **F** Heat map shows lncRNAs differentially expressed in the serum EVs of patients with NSCLC. **G** Volcano plot shows the dysregulated lncRNAs in EVs. **H** The levels of the top 45 most upregulated lncRNAs in the serum EVs of patients with NSCLC were detected by qPCR. **I** The dot plots show the relative levels of serum AL139294.1 in EVs in the healthy subset (*n* = 30) and the NSCLC subset (*n* = 30). **P < 0.01

To screen for differentially expressed lncRNAs in EVs, EVs were isolated from the serum of 3 healthy individuals and 3 patients with NSCLC. Western blotting was performed to detect specific proteins in EVs, namely, CD63, TSG101, and CD9, and EV-negative proteins [19], like calnexin and GAPDH (Fig. 1A). The expression of CD63, TSG101, CD9, calnexin, and GAPDH was evaluated in Beas-2B, NCI-H1299 and LTEP-A2 cells and EVs secreted by these cells (Fig. 1A). Scanning electron microscopy revealed that serum EVs derived from healthy individuals (Fig. 1B) and patients with NSCLC (Fig. 1C) exhibited typical cup-shaped morphological features. Additionally, the average diameters of serum EVs from healthy individuals (Fig. 1D) and patients with NSCLC (Fig. 1E) were 100 nm and 76 nm, respectively. The presence of proteins in EVs and the characteristic shape and size of EVs revealed the successful isolation of EVs from the serum of healthy individuals and patients with NSCLC. Subsequently, total RNA was extracted from serum EVs derived from 3 healthy individuals and 3 patients with NSCLC. LncRNA microarray analysis was performed to evaluate the lncRNA profiles of these EVs (Fig. 1F). A total of 496 differentially expressed lncRNAs were identified between healthy individuals and patients with NSCLC based on the screening criteria of *P*-values of < 0.05 and fold change values of > 2. Of the 496 lncRNAs, 146 lncRNAs were upregulated and 350 lncRNAs were downregulated in patients with NSCLC compared with healthy individuals (Fig. 1G). The top 45 significantly upregulated lncRNAs were selected based on smaller *P*-values and more considerable fold change values. qPCR was performed to quantify these lncRNAs in serum EVs derived from 10 patients with NSCLC. As shown in Fig. 1H, AL139294.1 had the smallest Cq value, indicating that it was most significantly upregulated in serum EVs derived from patients with NSCLC. Sanger sequencing revealed that the sequence of AL139294.1 amplicon, which was 153 bp in length, was consistent with that of AL139294.1 (Fig. S1A, B). Subsequently, the results of qPCR showed that the expression of AL139294.1 in EVs was significantly higher in the NSCLC group than in the healthy group (Fig. 1I). Therefore, we selected lncRNA AL139294.1 for subsequent analysis. We searched for the structure of AL139294.1 in the lnCAR database (https://lncar.renlab.org/) and found that AL139294.1 was 512-nt long and contained multiple stem-loops in the secondary structure (Fig. S1C). The expression of AL139294.1 was substantially higher in LUAD tissues (*n* = 535) than in normal tissues (*n* = 59) in the TCGA-LUAD dataset (Fig. S1D). However, it is unable to distinguish LUAD from healthy (*P* = 0.145, Fig. S1E). The cellular distribution of AL139294.1 was examined using the lncLocator database (http://www.csbio.sjtu.edu.cn/bioinf/lncLocator/). The results revealed that AL139294.1 was primarily localised in the cytoplasm, with a small proportion being encapsulated in EVs (Fig. S1F). These results suggest that AL139294.1 functions mainly in the cytoplasm and can be packaged into EVs.

### Effects of lncRNA AL139294.1 on lung cancer cells

**Fig. 2:**
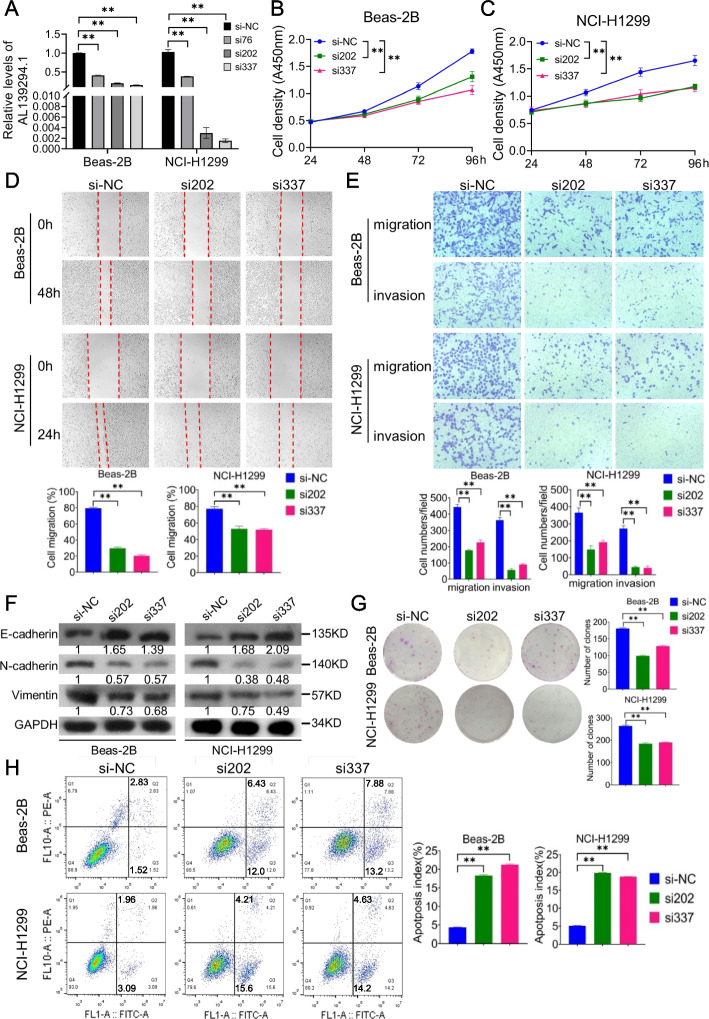
Knockdown of AL139294.1 inhibits the tumorigenic capacities of the NSCLC cells. **A** The levels of AL139294.1 in Beas-2B and NCI-H1299 cells were quantified after the transfection of si-NC, si76, si202, and si337. The effects of AL139294.1 knockdown on the proliferation ability of Beas-2B (**B**) and NCI-H1299 (**C**) cells were examined by CCK-8 assay. Wound healing assay (**D**) and transwell assay (**E**) were used to evaluate the migration and invasion abilities of Beas-2B and NCI-H1299 cells. **F** EMT-related markers were detected by western blotting in Beas-2B and NCI-H1299 cells after the knockdown of AL139294.1. **G** Colony formation assay was used to detect the clone formation ability of Beas-2B and NCI-H1299 cells after AL139294.1 knockdown. **H** Flow cytometry was performed to evaluate the effect of AL139294.1 knockdown on cells’ apoptosis. **P < 0.01

To investigate the role of AL139294.1 in the development of lung cancer, siRNAs (si76, si202, and si337) were used to knock down AL139294.1 in Beas-2B and NCI-H1299 cells. To determine transfection efficiency, the expression of AL139294.1 was evaluated in cells transfected with si76, si202, or si337. As shown in Fig. 2A, si202 and si337 significantly decreased the expression of AL139294.1. Therefore, we selected si202 and si337 for subsequent experiments. The results of the CCK-8 assay showed that AL139294.1 knockdown significantly inhibited the proliferative ability of Beas-2B (Fig. 2B) and NCI-H1299 (Fig. 2C) cells. The results of wound healing and transwell assays indicated that AL139294.1 knockdown remarkably weakened the migratory and invasive abilities of Beas-2B and NCI-H1299 cells (Fig. 2D, E). EMT represents an essential hallmark of metastasis. Therefore, western blotting was performed to evaluate the expression of EMT-related markers. AL139294.1 knockdown increased the expression of E-cadherin, an epithelial marker, but decreased the expression of N-cadherin and vimentin, mesenchymal markers (Fig. 2F), indicating that AL139294.1 affected cell migration and invasion by regulating EMT. Additionally, the colony formation assay demonstrated that AL139294.1 knockdown attenuated the colony-forming ability of Beas-2B and NCI-H1299 cells (Fig. 2G). Flow cytometry was used to examine the effects of AL139294.1 on cell apoptosis and cell cycle. The results showed that AL139294.1 knockdown significantly increased the apoptotic rate of Beas-2B and NCI-H1299 cells (Fig. 2H) and induced cell cycle arrest in G0/G1 phase (Fig. S2A and B). Altogether, these results indicate that AL139294.1 promotes the tumorigenic capacities of the NSCLC cell lines.

### Construction of the AL139294.1–miR-204-5p–BRD4 interaction axis

**Fig. 3:**
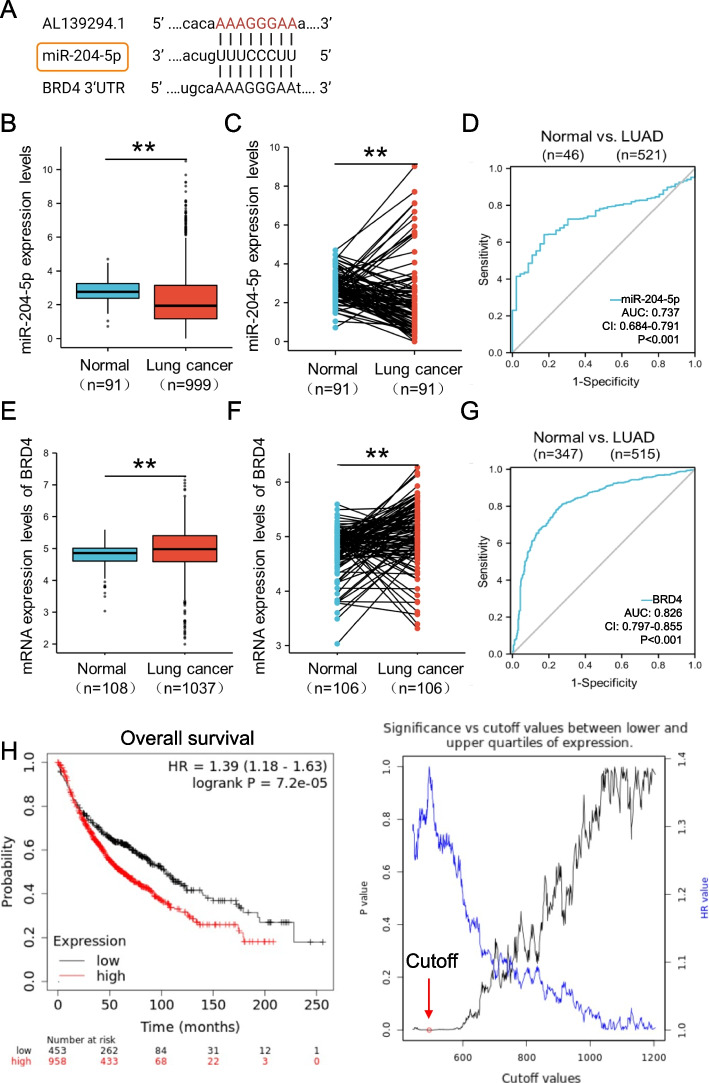
Bioinformatics analysis of the expression levels of miR-204-5p and BRD4. **A** The binding sites of AL139294.1 and miR-204-5p, as well as miR-204-5p and BRD4. TCGA cohort (TCGA-LUADLUSC dataset) shows lower levels of miR-204-5p in lung cancer tissues than those in normal tissues (**B**, normal, *n* = 91; lung cancer, *n* = 999) as well as paired tissues (**C**, normal, *n* = 91; lung cancer, *n* = 91). **D** ROC analyses were used to evaluate the diagnostic ability of miR-204-5p to distinguish normal (*n* = 46) and LUAD (*n* = 521) tissues in the TCGA cohort (TCGA-LUAD) dataset. Analyses of the TCGA cohort (TCGA-LUADLUSC dataset) show BRD4 mRNA levels in normal tissues (*n* = 108) and lung cancer tissues (**E**, *n* = 1037) as well as paired tissues (**F**, normal, *n* = 106; lung cancer, *n* = 106). **G** ROC analyses were used to estimate the diagnostic efficacy of BRD4 in distinguishing normal (*n* = 347) and LUAD (*n* = 515) tissues in the TCGA cohort and GTEx cohort (TCGA_GTEx-LUAD dataset). **H** Survival analyses (Kaplan–Meier plotter (kmplot.com)) show the association of BRD4 mRNA levels with overall survival (*n* = 1411) of patients with lung cancer. The cutoff is an auto-cutoff at which a significant difference is obtained between the lower and higher expression levels. The data were transformed by Log 2. **P < 0.01

A functional mechanism of lncRNAs is their interaction with miRNAs. Given that AL139294.1 was primarily localised in the cytoplasm (Fig. S1F), we investigated its mechanism from the perspective of ceRNAs. The sequence of AL139294.1 (Fig. S1A) was uploaded to miRDB (http://mirdb.org) to predict its target miRNAs (Fig. S3A). AL139294.1 was found to interact with miR-204-5p with a score of 61 (Fig. S3B). Because each miRNA has numerous target mRNAs, we screened for the target mRNAs of miR-204-5p in the miRWalk (http://mirwalk.umm.uni-heidelberg.de) and ENCORI (https://rnasysu.com/encori/) databases. Six hundred and five mRNA targets of miR-204-5p were found in both miRWalk and ENCORI (Table S4). Based on the published literature and a score of 0.92, BRD4 was selected as the target of miR-204-5p for further investigation (Fig. S3C). BRD4 is a transcriptional and epigenetic regulator that plays a pivotal role in embryogenesis and cancer development. After establishing the AL139294.1–miR-204-5p–BRD4 interaction network (Fig. 3A), we tried to analyse the expression patterns of the three RNAs in TCGA and CPTAC cohorts and examined their correlation with patient survival based on Kaplan–Meier curves. In TCGA cohort, the expression of miR-204-5p was considerably lower in lung cancer tissues than in normal tissues (Fig. 3B). Similar results could be found with the paired adjacent normal tissues and lung cancer tissues in TCGA cohort (Fig. 3C), with an AUC value of 0.737 to discriminate LUAD tissues from normal tissues (Fig. 3D). On the contrary, the mRNA expression of BRD4 was higher in lung cancer tissues than in normal tissues (Fig. 3E, F), with an AUC value of 0.826 to discriminate LUAD tissues from normal tissues (Fig. 3G). The upregulated mRNA expression of BRD4 was closely associated with poor overall survival (Fig. 3H), first-progression (Fig. S3D), and post-progression survival (Fig. S3E). In the CPTAC cohort, the protein expression pattern of BRD4 (Fig. S3F, G) was similar to its mRNA expression pattern in LUAD tissues and was positively associated with earlier tumour stages I and II (Fig. S3H). Altogether, these results suggest that dysregulated miR-204-5p and BRD4 are involved in the development of lung cancer.

### Role of miR-204-5p in lung cancer

**Fig. 4:**
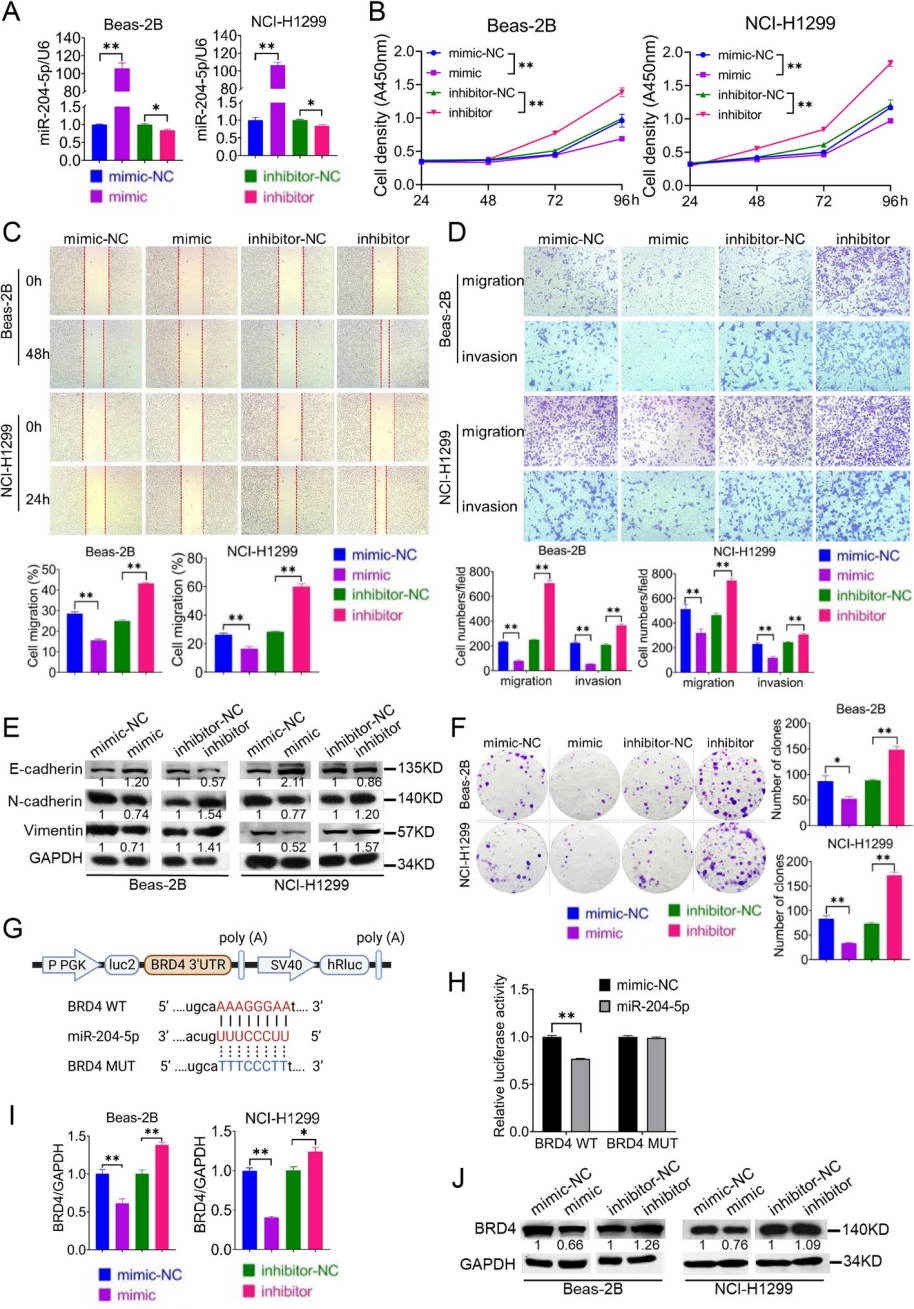
Role of miR-204-5p in the development of NSCLC. **A** The transfection efficiency of miR-204-5p mimics and inhibitors in Beas-2B and NCI-H1299 cells was checked by qPCR. **B** The proliferation ability of cells transfected with miR-204-5p mimics or inhibitors was measured by CCK-8 assay. Wound healing assay (**C**) and transwell assay (**D**) were used to measure the migration and invasion of NSCLC cells transfected with miR-204-5p mimics or inhibitors. **E** EMT-related markers were detected by western blotting after the knockdown or overexpression of miR-204-5p. **F** Colony formation assay was used to detect the clone formation ability of Beas-2B and NCI-H1299 cells after miR-204-5p knockdown or overexpression. **G** Schematic diagram shows the predicted binding sites of miR-204-5p with the 3’UTR of wild-type and mutant BRD4. **H** Luciferase reporter assay was conducted to test the binding of miR-204-5p with the 3’UTR of wild-type or mutant BRD4 in HEK-293 T cells. qPCR (**I**) and western blotting (**J**) were performed to detect BRD4 mRNA and protein levels after the transfection of miR-204-5p mimics or inhibitors, respectively. *P < 0.05, **P < 0.01

To examine the role of miR-204-5p and its interaction with BRD4 in NSCLC, miRNA mimics and inhibitors were used to upregulate and downregulate the expression of miR-204-5p, respectively, in Beas-2B and NCI-H1299 cells (Fig. 4A). Subsequently, CCK-8, wound healing, transwell and colony formation assays were performed to investigate the effects of miR-204-5p on lung cancer cells. The results showed that miR-204-5p mimics inhibited the proliferative, migratory, invasive, and colony-forming abilities of Beas-2B and NCI-H1299 cells, whereas miR-204-5p inhibitors promoted these abilities (Fig. 4B, C, D, F). miR-204-5p mimics increased the expression of E-cadherin and decreased the expression of N-cadherin and vimentin (Fig. 4E). However, miR-204-5p inhibitors had the opposite effects (Fig. 4E). Dual-luciferase reporter assay was performed to examine the direct interaction between miR-204-5p and BRD4. The results showed that miR-204-5p mimics reduced the luciferase activity of pmirGLO-BRD4-WT plasmids but not that of pmirGLO-BRD4-MUT plasmids (Fig. 4G, H). Additionally, miR-204-5p inhibitors increased the mRNA (Fig. 4I) and protein (Fig. 4J) expression of BRD4, whereas miR-204-5p mimics had the opposite effect. Altogether, these results suggest that miR-204-5p targets BRD4 to inhibit the proliferative, migratory, invasive, and colony-forming abilities of lung cancer cells.

### LncRNA AL139294.1 promotes the progression of NSCLC by indirectly regulating BRD4 and activating the Wnt and NF-κB2 pathways

**Fig. 5:**
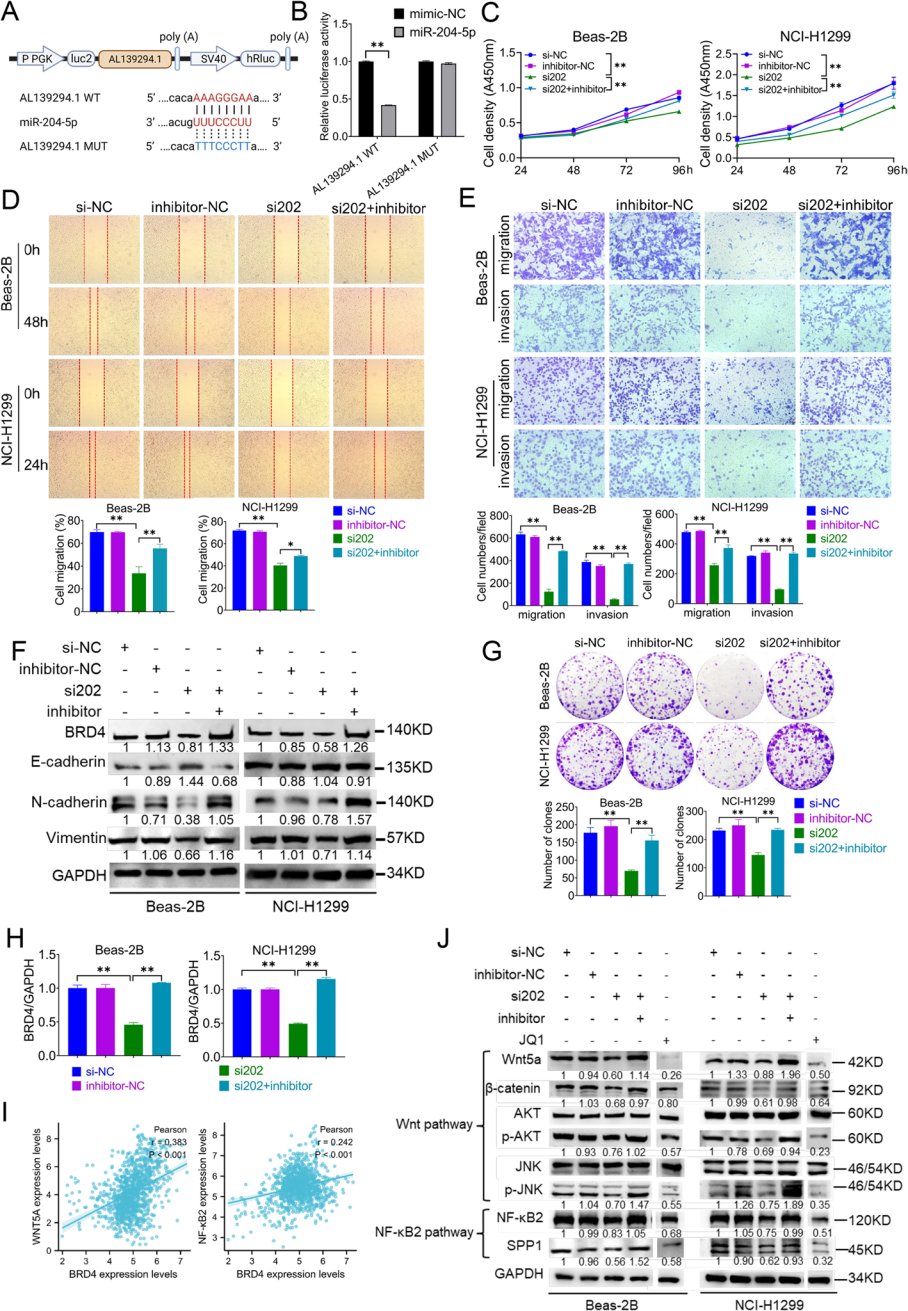
AL139294.1 promotes the tumorigenic capacities of the NSCLC cells by indirectly regulating BRD4 and activating the Wnt and NF-κB2 pathways. **A** Schematic diagram represents the predicted binding sites of miR-204-5p with wild-type and mutant AL139294.1. **B** Luciferase assay was conducted to examine the binding of miR-204-5p mimics with wild-type or mutant AL139294.1 in HEK-293T cells. **C** The effects of AL139294.1 knockdown, and the co-transfection of si202 with miR-204-5p inhibitors on the proliferation ability of Beas-2B and NCI-H1299 cells were examined by CCK-8 assay. Wound healing assay (**D**) and transwell assay (**E**) were used to evaluate the migration and invasion of Beas-2B and NCI-H1299 cells transfected with AL139294.1 si202 and miR-204-5p inhibitors. **F** Western blotting was carried out to test BRD4 and EMT-related proteins after the transfection of AL139294.1 si202 and miR-204-5p inhibitors. **G** Colony formation assay was performed to evaluate the colony formation ability of cells. **H** The mRNA levels of BRD4 in cells were detected by qPCR after the transfection of AL139294.1 si202 and miR-204-5p inhibitors. **I** TCGA-LUADLUSC dataset reveals the positive correlation of BRD4 mRNA with Wnt5a and NF-κB2 mRNAs in lung cancer tissues. **J** Western blotting was used to detect the levels of Wnt5a pathway-related proteins (Wnt5a, β-catenin, AKT, and JNK) and NF-κB2 pathway-related proteins (NF-κB2, and SPP1) in Beas-2B and NCI-H1299 cells treated with AL139294.1 si202, miR-204-5p inhibitors or JQ1. *P < 0.05, **P < 0.01

Dual-luciferase reporter assay was performed to examine the interaction of the AL139294.1–miR-204-5p–BRD4 axis in lung cancer. Overexpression of miR-204-5p significantly reduced the luciferase activity of wild-type AL139294.1 (Fig. 5A, B), verifying the direct binding of AL139294.1 with miR-204-5p. Subsequently, rescue experiments were performed to examine whether AL139294.1 affected NSCLC cells by interacting with miR-204-5p. The co-transfection of si202 targeting AL139294.1 and miR-204-5p inhibitors in Beas-2B and NCI-H1299 cells effectively attenuated the inhibitory effects of AL139294.1 knockdown on cell proliferation (Fig. 5C), wound healing (Fig. 5D), cell migration and invasion (Fig. 5E) and colony formation (Fig. 5G). The co-transfection reversed the AL139294.1 knockdown-induced increase in E-cadherin expression and decrease in N-cadherin and vimentin expression in the two cell lines (Fig. 5F). In addition, the co-transfection reversed the AL139294.1 knockdown-induced decrease in the mRNA (Fig. 5H) and protein (Fig. 5F) expression of BRD4, indicating that AL139294.1 regulated the expression of BRD4 through miR-204-5p and hence promoted NSCLC progression. We further detected the Wnt and NF-κB2 signaling pathways to elucidate the tumour-promoting mechanism of the AL139294.1–miR-204-5p–BRD4 axis in NSCLC. BRD4 mRNA was positively correlated with Wnt5a and NF-κB2 mRNAs in lung cancer tissues in the TCGA cohort (Fig. 5I). Western blotting was performed to investigate the effects of the AL139294.1–miR-204-5p–BRD4 axis on the expression of Wnt5a pathway-related proteins (Wnt5a, β-catenin, AKT and JNK) and NF-κB2 pathway-related proteins (NF-κB2 and SPP1). As shown in Fig. 5J, AL139294.1 knockdown by si202 decreased the protein expression of Wnt5a, β-catenin, pAKT, pJNK, NF-κB2 and SPP1 but that of non-phosphorylated AKT and non-phosphorylated JNK. However, the co-transfection of si202 and miR-204-5p inhibitors reversed this decrease. Similar to the effects of si202, the BRD4 inhibitor JQ1 downregulated the protein expression of Wnt5a, β-catenin, pAKT, pJNK, NF-κB2, and SPP1 (Fig. 5J), leading to inactivation of the Wnt and NF-κB2 pathways. We also carried out the co-transfection of si337 (the second siRNA targeting AL139294.1) and miR-204-5p inhibitors in Beas-2B and NCI-H1299 cells, and similar results could be found to those of the co-transfection of si202 and miR-204-5p inhibitors (Fig. S4). These results suggest that AL139294.1 promotes the progression of NSCLC through the miR-204-5p–BRD4–Wnt5a and miR-204-5p–BRD4–NF-κB2 pathways.

### LncRNA AL139294.1 promotes the progression of NSCLC through the miR-204-5p–BRD4–Wnt5a and miR-204-5p–BRD4–NF-κB2 pathways

**Fig. 6:**
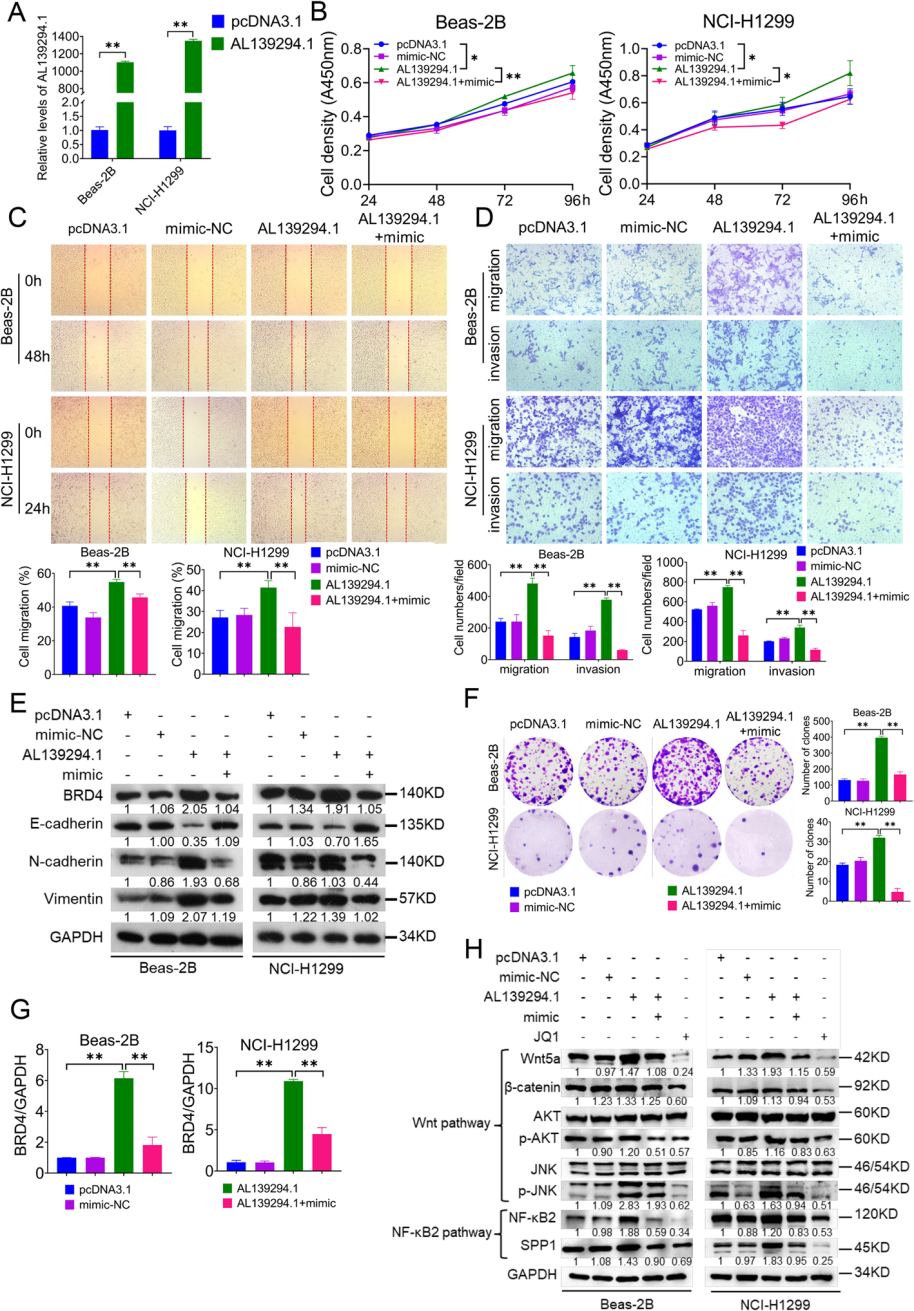
AL139294.1 promotes the tumorigenic capacities of the NSCLC cells through the miR-204-5p–BRD4–Wnt5a and miR-204-5p–BRD4–NF-κB2 pathways. **A** The levels of AL139294.1 in Beas-2B and NCI-H1299 cells were quantified after the transfection of pcDNA3.1 empty vectors or AL139294.1-overexpression plasmids. **B** The effects of AL139294.1 overexpression, miR-204-5p mimics, and their co-transfection on the proliferation ability of Beas-2B and NCI-H1299 cells were examined by CCK-8 assay. Wound healing assay (**C**) and transwell assay (**D**) were used to evaluate the migration and invasion of Beas-2B and NCI-H1299 cells transfected with AL139294.1-overexpression plasmids and miR-204-5p mimics. **E** Western blotting was carried out to detect BRD4 and EMT-related proteins after the transfection with AL139294.1-overexpression plasmids and miR-204-5p mimics. **F** Colony formation assay was performed to evaluate the colony formation ability of Beas-2B and NCI-H1299 cells. **G** The mRNA levels of BRD4 in cells were detected by qPCR after the transfection of AL139294.1-overexpression plasmids and miR-204-5p mimics. **H** Western blotting was used to test the levels of Wnt5a pathway-related proteins (Wnt5a, β-catenin, AKT, and JNK) and NF-κB2 pathway-related proteins (NF-κB2, and SPP1) in Beas-2B and NCI-H1299 cells treated with AL139294.1-overexpression plasmids, miR-204-5p mimics or JQ1. *P < 0.05, **P < 0.01

We constructed AL139294.1-overexpression plasmids and verified them via qPCR (Fig. 6A). Beas-2B and NCI-H1299 cells were transfected with AL139294.1-overexpression plasmids, and the combination of AL139294.1-overexpression plasmids and miR-204-5p mimics. As shown in Fig. 6, the results were consistent with those shown in Fig. 5. In particular, overexpression of AL139294.1 promoted cell proliferation (Fig. 6B), migration (Fig. 6C, D), invasion (Fig. 6D), EMT (Fig. 6E) and colony formation (Fig. 6F). It increased the mRNA (Fig. 6G) and protein (Fig. 6E) expression of BRD4 and the protein expression of Wnt5a, β-catenin, pAKT, pJNK, NF-κB2 and SPP1 (Fig. 6H). However, overexpression of miR-204-5p reversed the effects of AL139294.1 overexpression (Fig. 6B–H). Altogether, these results suggest that AL139294.1 upregulates BRD4 and activates the Wnt and NF-κB2 pathways by interacting with miR-204-5p, thereby leading to the development of NSCLC.

### Transport of lncRNA AL139294.1 by EVs and its role in NSCLC

**Fig. 7:**
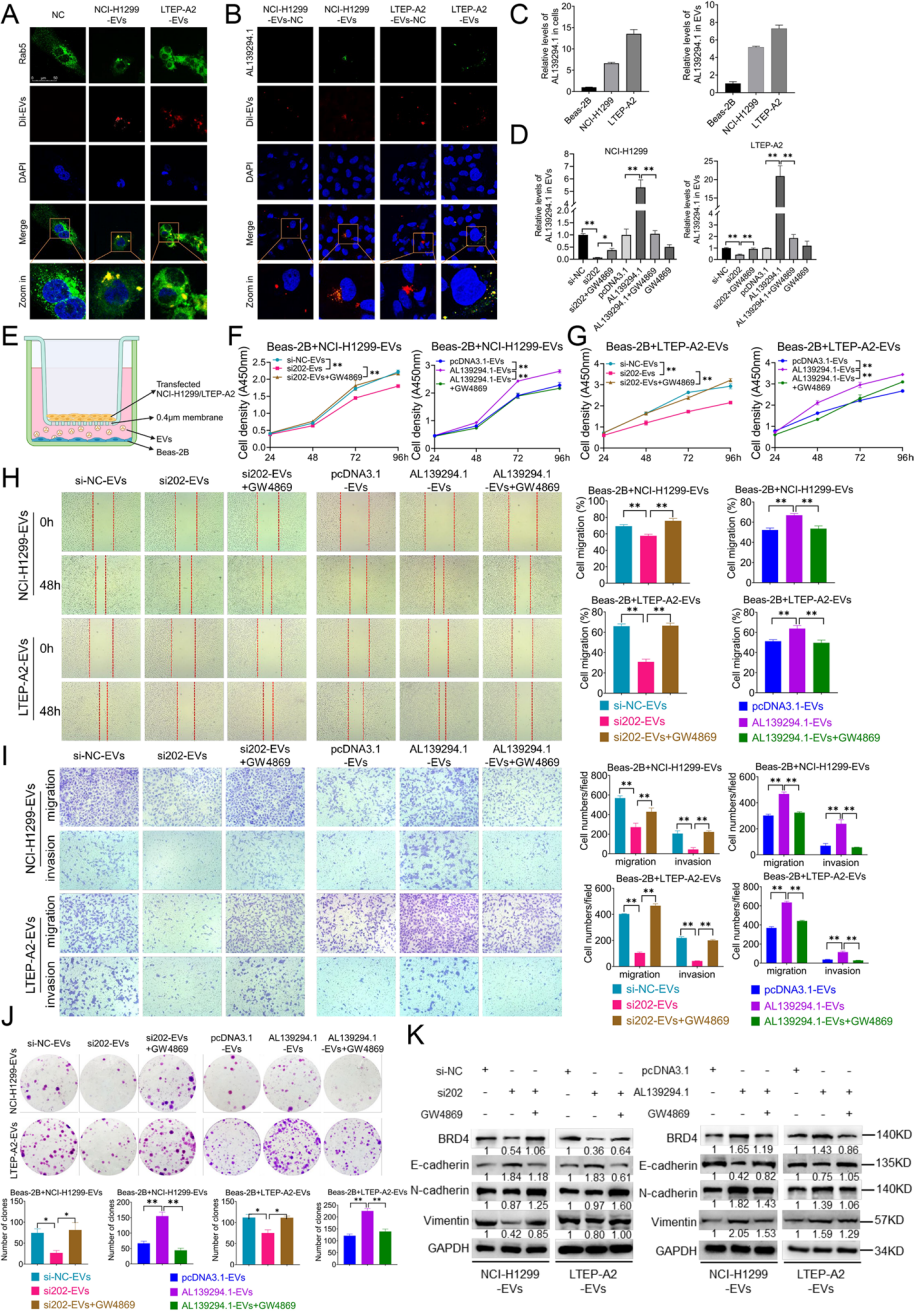
The transport of AL139294.1 by EVs and the effect of AL139294.1 in EVs on NSCLC. **A** Dil-labeled EVs (secreted by NCI-H1299 and LTEP-A2 cells, in red) and Beas-2B cells (transfected with Rab5 plasmids, in green) were co-cultured. The co-localization of EVs and Rab5 was photographed. **B** NCI-H1299 and LTEP-A2 cells were transfected with AL139294.1-overexpression plasmids with a GFP-tagged (in green), and the secreted EVs (in red) were co-cultured with Beas-2B cells. The co-localization of AL139294.1 and EVs in Beas-2B cells was imaged. **C** The levels of AL139294.1 in Beas-2B, NCI-H1299, LTEP-A2 cells, and their secreted EVs were quantified by qPCR. **D** The levels of AL139294.1 in secreted EVs from cells transfected with AL139294.1 si202 or overexpression plasmids were quantified by qPCR. **E** A co-culture system was used to study the effect of EVs secreted by treated NCI-H1299 or LTEP-A2 cells on Beas-2B cells. **F**, **G** CCK-8 assay was performed to evaluate Beas-2B cells’ proliferation ability. Wound healing assay (**H**) and transwell assay (**I**) were used to evaluate the migration and invasion ability of Beas-2B cells. **J** Colony formation assay was used to detect the clone formation ability of Beas-2B cells. **K** Western blotting was conducted to check the BRD4 and EMT-related proteins in Beas-2B cells after the co-culture. *P < 0.05, **P < 0.01

Because AL139294.1 was identified in serum EVs from patients with NSCLC, we hypothesised that it played an oncogenic role in recipient cells upon EV-mediated transport. To verify the hypothesis, we initially co-cultured Beas-2B cells with EVs derived from NCI-H1299 and LTEP-A2 cells and observed that the EVs were co-localised with endogenous Rab5 in the cytoplasm of recipient cells (Fig. 7A). Furthermore, AL139294.1-overexpression plasmids labeled with GFP were transfected into NCI-H1299 and LTEP-A2 cells. EVs were isolated from the respective cell culture media, stained, and co-cultured with Beas-2B cells. The results showed the co-localisation of AL139294.1-overexpression plasmids and EVs in the cytoplasm of Beas-2B cells, indicating that AL139294.1 were encapsulated in EVs from NCI-H1299 and LTEP-A2 cells and transported to recipient cells (Fig. 7B). To investigate the role of AL139294.1 transported by EVs in NSCLC, its expression was altered in EVs derived from NCI-H1299 and LTEP-A2 cells. As shown in Fig. 7C, the expression of AL139294.1 was substantially higher in NCI-H1299 and LTEP-A2 cells and EVs derived from these cells than in Beas-2B cells and EVs derived from Beas-2B cells. The transfection of si202 (Fig. 7D) but not si337 (data not shown) decreased the expression of AL139294.1 in EVs derived from NCI-H1299 and LTEP-A2 cells. On the contrary, the transfection of AL139294.1-overexpression plasmids increased the expression of AL139294.1 in EVs derived from NCI-H1299 and LTEP-A2 cells (Fig. 7D). Treatment with the EVs secretion inhibitor GW4869 (10 μM) reversed the effects of si202 and AL139294.1-overexpression plasmids (Fig. 7D). Therefore, we used si202 and AL139294.1-overexpression plasmids for further analysis. To investigate the influence of AL139294.1 in EVs on the function of recipient cells, Beas-2B cells were co-cultured with EVs derived from NCI-H1299 or LTEP-A2 cells transfected with either the AL139294.1-overexpression plasmid or siRNA (Fig. 7E). Downregulation or upregulation of AL139294.1 in EVs derived from NCI-H1299 cells inhibited or promoted cell proliferation, respectively (Fig. 7F). However, treatment with GW4869 attenuated the effects (Fig. 7F). Similar results were observed for EVs derived from transfected LTEP-A2 cells (Fig. 7G). Moreover, the EVs from the NCI-H1299 and LTEP-A2 cells transfected with si202 or AL139294.1-overexpression plasmids prevented or enhanced wound healing (Fig. 7H), migration (Fig. 7I), invasion (Fig. 7I) and colonization (Fig. 7J) abilities of Beas-2B cells, respectively. However, treatment with GW4869 reversed the effects (Fig. 7H–J). We continued to test the protein levels of EMT-related markers and BRD4. We found that the EVs from the NCI-H1299 and LTEP-A2 cells transfected with si202 or AL139294.1-overexpression plasmids increased or decreased the protein levels of E-cadherin, respectively, and GW4869 attenuated the influence in Beas-2B cells (Fig. 7K). At the same time, the EVs from the NCI-H1299 and LTEP-A2 cells transfected with si202 or AL139294.1-overexpression plasmids decreased or increased the protein levels of N-cadherin, Vimentin, and BRD4 in Beas-2B cells, respectively, and GW4869 rescued the effects (Fig. 7K). Altogether, these results suggest that AL139294.1 is transported by EVs to recipient cells and affects the oncogenic characteristics of the recipient cells by regulating BRD4.

### Effects of lncRNA AL139294.1 in EVs on tumour growth and the diagnostic value of AL139294.1 and miR-204-5p

**Fig. 8:**
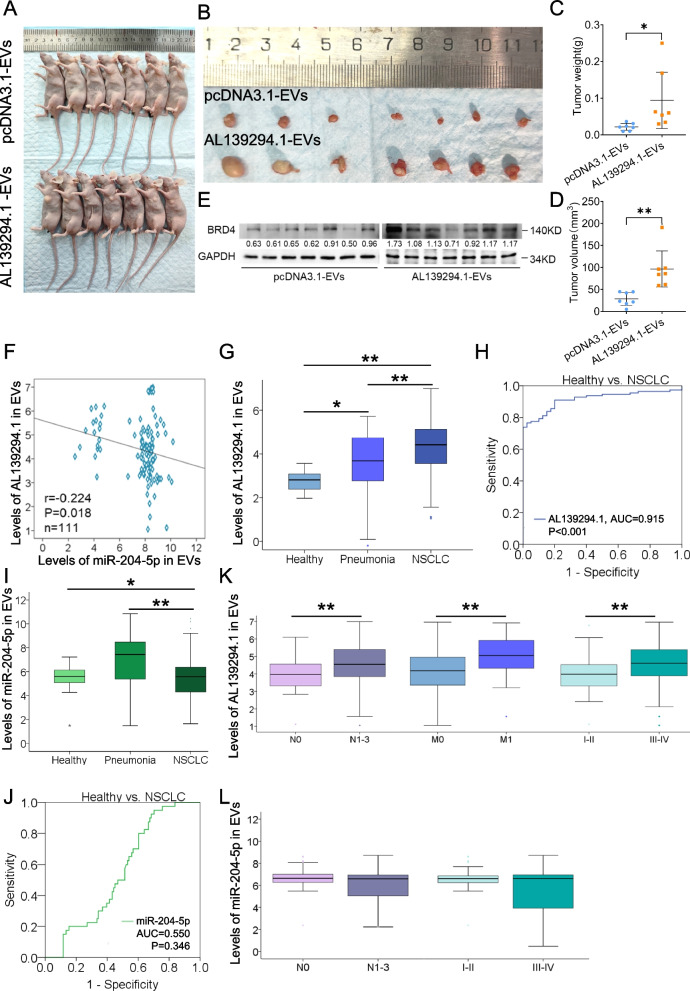
AL139294.1 in EVs promotes tumour growth in vivo and its diagnostic value. **A**, **B** Two groups of nude mice were subcutaneously injected with NCI-H1299 cells. After tumour formation, the pcDNA3.1-EVs or AL139294.1-EVs derived from NCI-H1299 cells transfected with pcDNA3.1 empty vectors or AL139294.1-overexpression plasmids, respectively, were injected into the nude mice via the lateral tail. Tumour weight (**C**) and volume (**D**) were measured. **E** The BRD4 protein levels in the tumour tissues were tested by western blotting. **F** The correlation of AL139294.1 and miR-204-5p in EVs of NSCLC (*n* = 111). The box plots show the relative levels of serum AL139294.1 (**G**) and miR-204-5p (**I**) in EVs of three cohorts: healthy (*n* = 40), pneumonia (*n* = 49), and NSCLC (*n* = 111). ROC analyses evaluated the diagnostic ability of serum AL139294.1 (**H**) and miR-204-5p (**J**) in EVs to differentiate healthy from NSCLC. **K** The box plots show the levels of serum AL139294.1 in EVs of the subgroups without/with lymph node metastases (N0/N1-3) (*n* = 38/73), without/with distant metastases (M0/M1) (*n* = 88/23), and stages I-II/III-IV (*n* = 44/67). **L** The box plots show the levels of serum miR-204-5p in EVs among the subgroups of without/with lymph node metastases (N0/N1-3) (*n* = 38/73) and stages I-II/III-IV (*n* = 44/67). *P < 0.05, **P < 0.01

To examine the effect of AL139294.1 in EVs on tumour growth, subcutaneous xenograft tumour models were established using NCI-H1299 cells. Injection of EVs derived from NCI-H1299 cells into mice increased tumour weight and volume in the AL139294.1-overexpression group when compared with the control group (Fig. 8A–D). Western blotting showed that overexpression of AL139294.1 in EVs derived from NCI-H1299 cells elevated the protein expression of BRD4 in tumour tissues (Fig. 8E). After validating the oncogenic effect of AL139294.1 in EVs on NSCLC through in vitro and in vivo experiments, we examined its diagnostic value using serum samples from healthy individuals, patients with pneumonia and patients with NSCLC. The serum levels of AL139294.1 and miR-204-5p in EVs were evaluated via qPCR. The expression of AL139294.1 in EVs was negatively correlated with that of miR-204-5p in the serum samples of the NSCLC group (*r* = -0.224, *P* = 0.018, Fig. 8F) but not in those of the other groups (data not shown). The serum levels of AL139294.1 in EVs were significantly higher in the NSCLC group than in the healthy and pneumonia groups (Fig. 8G), with an AUC value of 0.915 to differentiate healthy samples from NSCLC samples (Fig. 8H). In addition, the serum levels of miR-204-5p in EVs were lower in the NSCLC group than in the healthy and pneumonia groups (Fig. 8I) but showed poor ability (AUC = 0.550, *P* = 0.346) to distinguish healthy samples from NSCLC samples (Fig. 8J). Furthermore, AL139294.1 and miR-204-5p in EVs were not found to be associated with age, sex, tumour size, and histological subtype (data not shown). However, upregulated AL139294.1 in EVs was closely associated with lymph node metastasis, distant metastasis, and advanced stages (Fig. 8K), whereas downregulated miR-204-5p in EVs was associated with lymph node metastasis and advanced stages (Fig. 8L). Altogether, these results indicate that the serum levels of AL139294.1 and miR-204-5p in EVs have diagnostic value in NSCLC, particularly more aggressive NSCLC.

Regarding the quantification of AL139294.1 in EVs, there is no consistency in the selection of reference genes for data normalization. In the present study, we chose GAPDH as the house-keeping genes based on literature, our previous studies, and the validation in a small sample cohort. Alternatively, an absolute data normalization strategy based on standard curves, and the emerging digital PCR technique may help researchers quantify the copy numbers of ncRNAs in EVs.

We also quantified AL139294.1 and miR-204-5p in the serum samples. As shown in Fig. S5, the serum levels of AL139294.1 and miR-204-5p show similar expression patterns to the levels of serum AL139294.1 and miR-204-5p in EVs among the cohorts of healthy, pneumonia and NSCLC. However, serum AL139294.1 shows a lower diagnostic ability (AUC = 0.851) than AL139294.1 in EVs (AUC = 0.915, Fig. 8H) to discriminate healthy individuals from patients with NSCLC. Additionally, the levels of serum AL139294.1 show no differences between the subsets of N0 and N1-3, M0 and M1, and tumour stages I-II and III-IV. Sed between the subgroups of N0 and rum miR-204-5p shows a higher diagnostic ability (AUC = 0.857) than miR-204-5p in EVs (AUC = 0.550, Fig. 8J) to discriminate healthy persons from patients with NSCLC, but no obvious differences could be founN1-3, M0 and M1, and tumour stages I-II and III-IV. These results indicate that serum AL139294.1 in EVs, but not serum AL139294.1 is closely related to advanced tumour characteristics, showing superior diagnostic potential for NSCLC.

**Fig. 9:**
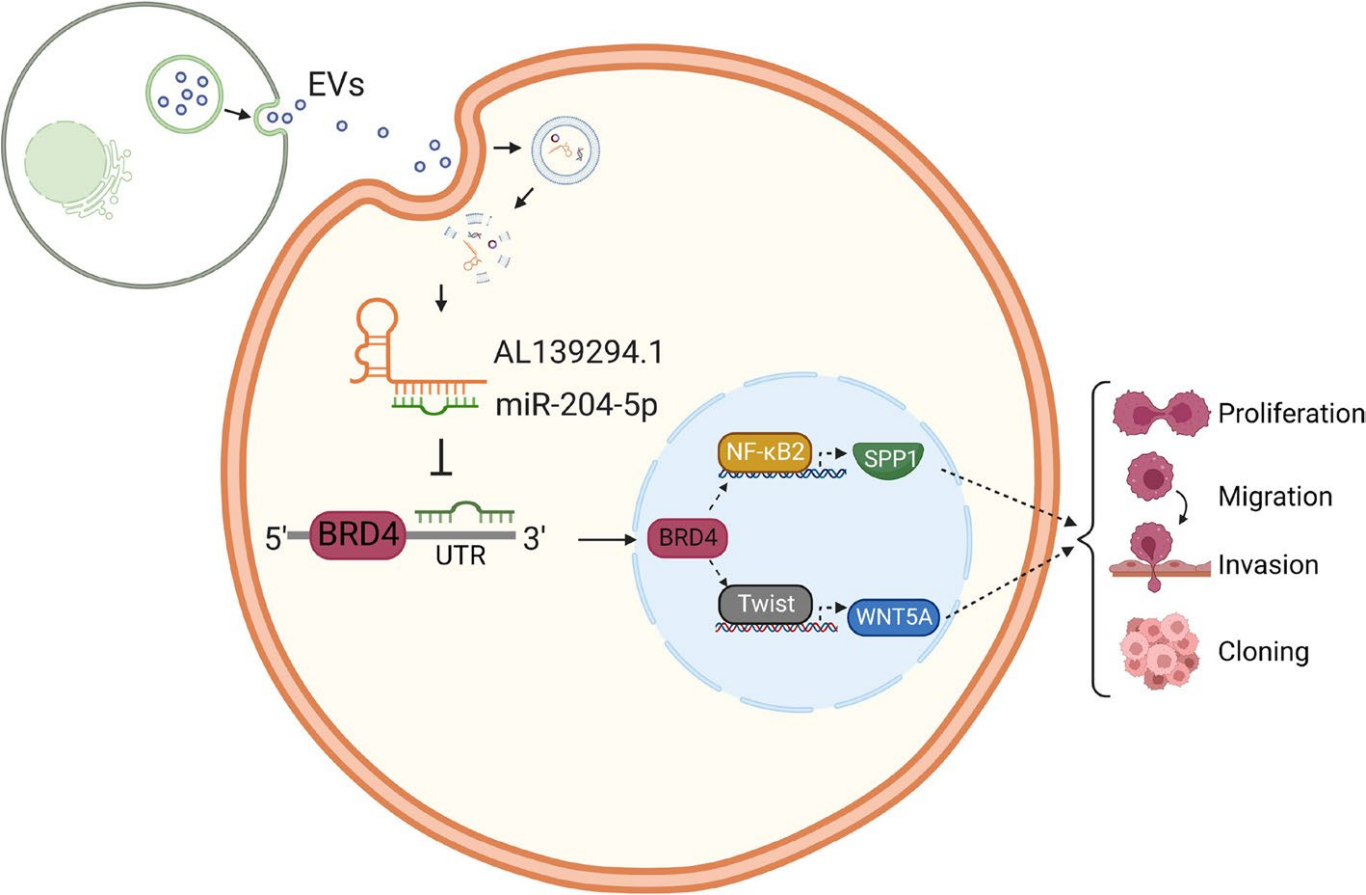
Schematic diagram. EVs carrying AL139294.1 are secreted by the origin cells and enter the recipient cells. In the cytoplasm of recipient cells, AL139294.1 is released. It interacts with miR-204-5p, leading to the derepressing of miR-204-5p on its target BRD4, which further activates the Wnt5a and NF-κB2 signaling pathways and finally contributes to the progression of NSCLC

Collectively, AL139294.1 could be packaged into EVs. After its transport to recipient cells by EVs, AL139294.1 sponged miR-204-5p and thereby weakened the inhibitory effect of miR-204-5p on BRD4, further activating the Wnt and NF-κB2 pathways, which promoted the proliferative, migratory, and invasive abilities of the recipient cells (Fig. 9).

## Discussion

EVs play a crucial role in tumorigenesis via cell-to-cell communication. LncRNAs are a crucial component of EVs. Tumour-associated lncRNAs can be packaged into EVs and transported to recipient cells. Consequently, these lncRNAs in EVs influence the proliferative, migratory, and invasive abilities of recipient cells and their response to chemotherapy [20]. Li et al. [21] found that upregulated lncRNA Sox2ot in plasma-derived EVs regulated the expression of SRY-box transcription factor 2 (Sox2) by sponging the miR-200 family, thereby promoting the metastasis of pancreatic ductal adenocarcinoma (PDAC). In addition, upregulated Sox2ot in EVs was closely associated with prognosis in PDAC [21]. Zang et al. [22] identified the NSCLC-related lncRNA UFC1 in the serum EVs of patients with NSCLC and demonstrated that these EVs transported UFC1 to promote NSCLC progression via epigenetic silencing of PTEN. In this study, we identified lncRNA AL139294.1 in the serum EVs of patients with NSCLC based on lncRNA microarray analysis and examined its oncogenic role in NSCLC (Fig. 2). Additionally, we found that AL139294.1 was transported to recipient cells by EVs and established a co-culture system to examine the oncogenic effect of AL139294.1 in EVs on recipient cells (Fig. 7). AL139294.1 is 512-nt long and contains multiple stem-loops in the secondary structure. To the best of our knowledge, no studies have investigated the role of AL139294.1 in cancers or other diseases. As vital epigenetic regulators, lncRNAs regulate gene expression mainly by interacting with proteins or miRNAs. Based on the ceRNA theory and the localisation of AL139294.1 in the cytoplasm, we established the AL139294.1–miR-204-5p–BRD4 interaction axis to demonstrate the functional mechanism of AL139294.1 in the progression of NSCLC. MiR-204-5p has been reported to be involved in the metastasis of various cancers, such as breast cancer [23], oesophageal squamous cell carcinoma [24], oral squamous cell carcinoma [25], and prostate cancer [26]. Wa et al. [26] found that overexpression of miR-204-5p inhibited the migratory and invasive abilities of prostate cancer cells in vitro and repressed bone metastasis of prostate cancer in vivo. Therefore, miR-204-5p is frequently considered a tumour suppressor. Given that each miRNA can target multiple mRNAs by binding to their 3’-UTRs, we selected BRD4 as the target of miR-204-5p for further investigation. The results showed that miR-204-5p directly interacted with AL139294.1 and BRD4. BRD4 is a member of the bromodomain and extraterminal domain protein family [27]. BRD4 is a well-known tumour-associated gene, and its upregulation is closely related to the development of various cancers, including lung cancer [28, 29]. It can interact with Twist to regulate the expression of Wnt5a, a ligand of the Wnt pathway, thus contributing to the development and metastasis of breast cancer [30]. In addition, BRD4 promotes the progression of melanoma by regulating the expression of secreted phosphoprotein 1 (SPP1) through the nuclear factor-kappa B2 (NF-κB2) pathway [31]. Numerous small-molecule inhibitors targeting BRD4, such as BMS-986158, OTX-015, JQ1, and GSK-525762 [32, 33], have been synthesised and used for treating cancers in clinical settings. Upon its transport to recipient cells, AL139294.1 promoted the proliferative, migratory, and invasive abilities of the cells by activating the Wnt and NF-κB2 pathways (Figs. 5, 6, 7, and 9). In addition, AL139294.1 in EVs promoted tumour growth in vivo. To examine the oncogenic role of AL139294.1 from the perspective of protein interaction, StarBase (https://starbase.sysu.edu.cn/index.php) and catRAPID (http://s.tartaglialab.com/page/catrapid_group) were used to predict proteins that interact with AL139294.1. However, no potential interacting proteins have been reported to date.

The ceRNA regulatory network is complicated. AL139294.1 might interact with several miRNAs, and each miRNA could target multiple mRNAs. In the present study, we chose BRD4 as the starting point to construct the AL139294.1–miR-204-5p–BRD4 interaction axis, because BRD4 is a well-known tumour-associated gene, and its upregulation is closely related to the development of various cancers. Currently, there are many commercially available small-molecule inhibitors targeting BRD4, such as BMS-986158, OTX-015, JQ1, and GSK-52576 to treat cancers in the clinic. The understanding of the regulatory mechanism of BRD4 might help guide medication and predict treatment response.

Furthermore, we examined the clinical significance of AL139294.1 and miR-204-5p in EVs as markers for diagnosing NSCLC. A negative correlation was observed between the two RNAs in the NSCLC cohort (*r* = -0.224, *P* = 0.018, Fig. 8F). Remarkably, the diagnostic performance of AL139294.1 in EVs was superior to that of miR-204-5p in EVs (Fig. 8). The high expression of AL139294.1 in EVs and low expression of miR-204-5p in EVs were strongly associated with more aggressive features of NSCLC (Fig. 8).

Altogether, the findings of this study elucidate the mechanism through which upregulated AL139294.1 in EVs promotes the progression of NSCLC and highlight the diagnostic significance of AL139294.1 in EVs for NSCLC. However, further studies are warranted to examine the cause of the upregulation of AL139294.1 and its selective encapsulation into EVs during the development of NSCLC. In addition, large-sample studies with a long-term follow-up should be conducted to verify the diagnostic value of AL139294.1 and miR-204-5p in EVs.

In conclusion, NSCLC-related lncRNA AL139294.1 can be packaged into EVs and transported to recipient cells. The transported AL139294.1 competitively binds to miR-204-5p to regulate BRD4 and activate the Wnt and NF-κB2 pathways, consequently promoting the proliferative, migratory, and invasive abilities of recipient cells. In addition, AL139294.1 and miR-204-5p in EVs can be used as diagnostic markers for NSCLC, particularly more aggressive NSCLC. Therefore, the novel lncRNA AL139294.1 in EVs identified in this study is a promising target for the diagnosis and treatment of lung cancer.

### Supplementary Information

**Additional file 1:** **Supplementary Table 1.** The sequences of control, siRNA, miR-204-5p mimics, miR-204-5p inhibitors, and plasmid construction primers in the present study.

**Additional file 2:** **Supplementary Table 2.** Antibodies used in the experiments.

**Additional file 3: Supplementary Table 3.** The sequences of primers in the present study.

**Additional file 4. **

**Additional file 5: Fig. S1.** The structure and cellular localization of AL139294.1. A The sequence of AL139294.1. B Sanger sequencing verified the specificity of the AL139294.1 amplification product. C The lnCAR database (https://lncar.renlab.org/) shows that AL139294.1 is 512 nt long, and its secondary structure contains multiple stem loops. D TCGA cohort (TCGA-LUAD dataset) shows the levels of AL139294.1 in normal (*n* = 59) and LUAD tissues (*n* = 535). E ROC analysis was performed to estimate the diagnostic efficacy of AL139294.1 in distinguishing normal (*n* = 59) and LUAD (*n* = 535). F The cellular location of AL139294.1 was checked by the lncLocator database (http://www.csbio.sjtu.edu.cn/bioinf/lncLocator/). *P < 0.05.

**Additional file 6: Fig. S2. **The effect of AL139294.1 knockdown on cells’ cycle. Flow cytometry detected the effect of AL139294.1 knockdown on Beas-2B (A) and NCI-H1299 (B) cell cycle. *P < 0.05 and **P < 0.01.

**Additional file 7: Fig. S3. **Prediction of AL139294.1–miR-204-5p–BRD4 regulatory axis. A The miRDB database (http://mirdb.org) was used to predict miRNAs that interact with AL139294.1. B MiR-204-5p was found to have the binding site with AL139294.1. C miRWalk database (http://mirwalk.umm.uni-heidelberg.de) was used to predict the binding sites of miR-204-5p and BRD4. Survival analyses (Kaplan-Meier plotter (kmplot.com)) show the association of BRD4 mRNA levels with first-progression (D, *n* = 874), and post-progression survival (E, *n* = 242) of patients with lung cancer. The cutoff is an auto-cutoff at which a significant difference is obtained between the lower and higher expression levels. F, G CPTAC cohort (LUAD dataset) analyses present higher protein levels of BRD4 in LUAD tissues (*n* = 109) than those in normal tissues (*n* = 102) as well as paired tissues (normal, *n* = 102; LUAD, *n* = 102). H The protein levels of BRD4 are different in normal tissues (*n* = 11) and LUAD tissues with stages 1 (*n* = 59), 2 (*n* = 30), 3 (*n* = 21), and 4 (*n* = 1) based on the CPTAC cohort (LUAD dataset). The data were transformed by Log 2. **P < 0.01.

**Additional file 8: Fig. S4. **AL139294.1 promotes the tumorigenic capacities of the NSCLC cells by indirectly regulating BRD4 and activating the Wnt and NF-κB2 pathways. The effects of AL139294.1 knockdown, and the co-transfection of si337 and miR-204-5p inhibitors on the proliferation ability of Beas-2B (A) and NCI-H1299 (B) cells were examined by CCK-8 assay. Wound healing assay (C) and transwell assay (D) were used to evaluate the migration and invasion of Beas-2B and NCI-H1299 cells transfected with AL139294.1 si337 and miR-204-5p inhibitors. E Western blotting was carried out to test BRD4 and EMT-related proteins after the transfection of AL139294.1 si337 and miR-204-5p inhibitors. F Colony formation assay was performed to evaluate the colony formation ability of cells. G The mRNA levels of BRD4 in cells were detected by qPCR after the transfection of AL139294.1 si337 and miR-204-5p inhibitors. H Western blotting was used to detect the levels of Wnt5a pathway-related proteins (Wnt5a, β-catenin, AKT, and JNK) and NF-κB2 pathway-related proteins (NF-κB2, and SPP1) in Beas-2B and NCI-H1299 cells treated with AL139294.1 si337, miR-204-5p inhibitors or JQ1. *P < 0.05, **P < 0.01.

**Additional file 9: Fig. S5.** The levels of serum AL139294.1 and miR-204-5p. The box plots show the relative levels of serum AL139294.1 (A) and miR-204-5p (F) in three cohorts: healthy (*n* = 40), pneumonia (*n* = 49), and NSCLC (*n* = 111). ROC analyses evaluated the diagnostic ability of serum AL139294.1 (B) and miR-204-5p (G) to differentiate healthy from NSCLC. The box plots show the levels of serum AL139294.1 (C, D, E) and miR-204-5p (H, I, J) in the subgroups of N0 (*n*=38) and N1-3 (*n* = 73), M0 (*n* = 88) and M1 (*n* = 23), and stages I-II (*n*=44) and III-IV (*n* = 67). **P < 0.01.

## Data Availability

The datasets used and/or analyzed during the current study are available from the corresponding author on reasonable request.

## Abbreviations

LncRNA: Long non-coding RNA
NSCLC: Non-small-cell lung cancer
BRD4: Bromodomain-containing protein 4
LUAD: Lung adenocarcinoma
LUSC: Lung squamous cell carcinoma
MiRNA: MicroRNA
CircRNA: Circular RNA
mRNA: Messenger RNAs
EMT: Epithelial–mesenchymal transition
CeRNAs: Competitive endogenous RNAs
Sox2: SRY-Box transcription factor 2
PDAC: Pancreatic ductal adenocarcinoma
CCK-8: Cell counting kit-8
PBS: Phosphate buffer saline
TCGA: The Cancer Genome Atlas
CPTAC: The Clinical Proteomic Tumor Analysis Consortium
ROC: Receiver operating characteristic
AUC: Areas under the curves
