# Supplementary material for: LncRNA AL139294.1 can be transported by extracellular vesicles to promote the oncogenic behaviour of recipient cells through activation of the Wnt and NF-κB2 pathways in non-small-cell lung cancer
Source: J Exp Clin Cancer Res. 2024 Jan 16;43:20. doi: 10.1186/s13046-023-02939-z (PMC10790371; doi:10.1186/s13046-023-02939-z)
Supplement: Supplementary file 1 — Additional file 1: Supplementary Table 1. The sequences of control, siRNA, miR-204-5p mimics, miR-204-5p inhibitors, and plasmid construction primers in the present study. [file 13046_2023_2939_MOESM1_ESM.docx]

Supplementary Table 1. The sequences of control, siRNA, miR-204-5p mimics, miR-204-5p inhibitors, and plasmid construction primers in the present study

| Gene | sequences (5’ to 3’) | |
| --- | --- | --- |
| si-NC | sense | UUCUCCGAACGUGUCACGUTT |
|  | antisense | ACGUGACACGUUCGGAGAATT |
| si76 | sense | GCUAAGAGAUCAAGGACAUTT |
|  | antisense | AUGUCCUUGAUCUCUUAGCTT |
| si202 | sense | CCUAGAGUCAGAGGAAGCUTT |
|  | antisense | AGCUUCCUCUGACUCUAGGTT |
| si337 | sense | CCAGGAUGACACUACCAAUTT |
|  | antisense | AUUGGUAGUGUCAUCCUGGTT |
| mimic-NC | sense | UUCUCCGAACGUGUCACGUTT |
|  | antisense | ACGUGACACGUUCGGAGAATT |
| miR-204-5p mimics | sense | UUCCCUUUGUCAUCCUAUGCCU |
|  | antisense | GCAUAGGAUGACAAAGGGAAUU |
| inhibitor-NC | sense | CAGUACUUUUGUGUAGUACAA |
| miR-204-5p inhibitor | sense | AGGCAUAGGAUGACAAAGGGAA |
| lncRNA AL139294.1 | sense | AAGCTTACCCCATCCCCTTCATACAC |
|  | antisense | GGATCCTGGTTTGGAAATCCAGAATTTAATATTAAAATACAC |
