# Supplementary material for: LncRNA AL139294.1 can be transported by extracellular vesicles to promote the oncogenic behaviour of recipient cells through activation of the Wnt and NF-κB2 pathways in non-small-cell lung cancer
Source: J Exp Clin Cancer Res. 2024 Jan 16;43:20. doi: 10.1186/s13046-023-02939-z (PMC10790371; doi:10.1186/s13046-023-02939-z)
Supplement: Supplementary file 2 — Additional file 2: Supplementary Table 2. Antibodies used in the experiments. [file 13046_2023_2939_MOESM2_ESM.docx]

Supplementary Table 2. Antibodies used in the experiments.

| Antibody | Catalog # | Supplier | Dilution |
| --- | --- | --- | --- |
| CD63 | AP5333b-ev | ABGENT | 1:1000 |
| CD9 | AP1482d-ev | ABGENT | 1:1000 |
| TSG101 | A2216 | Abclonal | 1:1000 |
| Calnexin | A4846 | Abclonal | 1:1000 |
| E-cadherin | 3195S | CST | 1:1000 |
| N-cadherin | 13116S | CST | 1:1000 |
| Vimentin | bs-0756R | Bioss | 1:1000 |
| BRD4 | A12677 | Abclonal | 1:1000 |
| β-catenin | A0316 | Abclonal | 1:1000 |
| Wnt5a | A19133 | Abclonal | 1:1000 |
| AKT | A17909 | Abclonal | 1:1000 |
| p-AKT | AF0016 | Affinity | 1:1000 |
| JNK | A4867 | Abclonal | 1:1000 |
| p-JNK | AF3318 | Affinity | 1:1000 |
| NF-κb2 | A19605 | Abclonal | 1:1000 |
| SPP1 | A1361 | Abclonal | 1:1000 |
| GAPDH | T0004 | Affinity | 1:5000 |
| HRP-anti-mouse | 115-035-003 | jackson immunoresearch | 1:5000 |
| HRP-anti-rabbit | 111-035-003 | jackson immunoresearch | 1:5000 |
| IRDye® 680RD Goat anti-Rabbit | 925-68071 | LI-COR | 1:10000 |
| IRDye® 800CW Goat anti-Mouse | 926-32210 | LI-COR | 1:10000 |
