# Supplementary material for: LncRNA AL139294.1 can be transported by extracellular vesicles to promote the oncogenic behaviour of recipient cells through activation of the Wnt and NF-κB2 pathways in non-small-cell lung cancer
Source: J Exp Clin Cancer Res. 2024 Jan 16;43:20. doi: 10.1186/s13046-023-02939-z (PMC10790371; doi:10.1186/s13046-023-02939-z)
Supplement: Supplementary file 3 — Additional file 3: Supplementary Table 3. The sequences of primers in the present study. [file 13046_2023_2939_MOESM3_ESM.docx]

Supplementary Table 3. The sequences of primers in the present study

| Gene | Primer (5’ to 3’) | | Annealing temperature |
| --- | --- | --- | --- |
| LINC02428 | forward | TGTCTGGAATAGCAAATTGACCT | 60°C |
|  | reverse | ACTCAGAGAACAACTAATTTCAGC |  |
| AP001604.1 | forward | GCCGAAACTACTCTCTCTCCC | 60°C |
|  | reverse | TGCACCTAACAGAGTGGTGG |  |
| AC009480.1 | forward | GAATGACAAATGTTATGGCACTGT | 60°C |
|  | reverse | AGTCTTGCTGCGGATTTTTGG |  |
| AC009065.1 | forward | GTGAGTCTAGGAGTGGCCTG | 60°C |
|  | reverse | CTTGTGATGTTGTGGCAGTGG |  |
| AL591503.1 | forward | TAGGACATTTCCATAAGTTCTGGGT | 60°C |
|  | reverse | GGTTGGGCACTCTTGGTCAC |  |
| AL136088.1 | forward | TCTCCCCCTCTTCTGACATCTT | 60°C |
|  | reverse | GATGCTGCAAACAAACACAAACA |  |
| AL139294.1 | forward | TGTCACAGCAGATGCCACAT | 60°C |
|  | reverse | CCCACTCGCTGCCTATAACA |  |
| LINC02336 | forward | GAGGTTCCTGACCAAGGGGA | 60°C |
|  | reverse | AGCGCTTTATGTGCAGGAAA |  |
| AC107396.1 | forward | GGGGCTTGAATGGCAAAGATG | 60°C |
|  | reverse | CCCTTGAGACACTTAGTGCAGA |  |
| IGBP1-AS2 | forward | TCTCCTGGCGAGATGAGTTAGA | 60°C |
|  | reverse | AGCCCGTATGGTATGTTGGG |  |
| GRK5-IT1 | forward | CTTCACAGGCCAGCAGTAGC | 60°C |
|  | reverse | TTGCTAGGGCCTCAGGAAAAT |  |
| AC016876.1 | forward | CCCTCGAGAGGAGCCCTG | 60°C |
|  | reverse | GTTCGTCGCGTTTCCTTCAC |  |
| AC073336.1 | forward | AGGTATGAGCCACTGGCATT | 60°C |
|  | reverse | CAACAGCACATCTGGTTGGC |  |
| AC013724.1 | forward | GCTTGGAGGCTAAGAGTTCCC | 60°C |
|  | reverse | CTCGTTTCAGGGCTGGCTAT |  |
| AC097487.1 | forward | AACCTGGAAAGCAGGCAGATAA | 60°C |
|  | reverse | TTTCCCAAGCACGAAGAAGTT |  |
| LINC01438 | forward | TGGTGCATAACAGCCGTTTC | 60°C |
|  | reverse | AAGAGCTGTTCTGGTGTCCTG |  |
| AP003096.1 | forward | TGAGTTGGGCTGTGATGAGC | 60°C |
|  | reverse | CCACCTCTACATGCGAGTCC |  |
| LINC02388 | forward | AAGATCCCATGACAAGACAGCTC | 60°C |
|  | reverse | CAGACACACCAGAGGTTTCAGA |  |
| AC093010.2 | forward | AGCGAGCGAATGAGCAAGAG | 60°C |
|  | reverse | GCAGTGCCGAGACTTATGGA |  |
| BX324167.1 | forward | ACACAGCAGCCTCATCTTCT | 60°C |
|  | reverse | TCTCTGGACATTGAGGCTCC |  |
| AL158055.1 | forward | AGGTGTGGGGTGAAGAGTTG | 60°C |
|  | reverse | CAGAAGCGATTGTGGATGTGT |  |
| AC010980.2 | forward | GAGATCCTGAGCGCTGAGAG | 60°C |
|  | reverse | AACCACAAGCTTCCAGGAGA |  |
| AC018511.1 | forward | TGCTGGGATTATAGGCGTGA | 60°C |
|  | reverse | TGGATTAAAGAGGGCAGGCA |  |
| AC009716.1 | forward | ACTCAAGTTAACAGGGGCCA | 60°C |
|  | reverse | GTTGCTAGCTTTCCTCCTGC |  |
| AL136084.3 | forward | GGCTGCCTTATGTAACCTGC | 60°C |
|  | reverse | GCCTCAGTCTCCTTGTCCAT |  |
| AC006262.3 | forward | ACGACGGCATCATCATAGCA | 60°C |
|  | reverse | CGAAGCCTGGGACATGTAGG |  |
| AC025278.1 | forward | AGAGCGAGACATCTCAGTGC | 60°C |
|  | reverse | ACACGGACCTCTCCACTCAA |  |
| AC010997.5 | forward | GATTGGGGGCCGGTTCCTTT | 60°C |
|  | reverse | CTGTCAAAACCCAGTCATCGC |  |
| AC096666.1 | forward | GCGATATGCAGAAGGTCCGT | 60°C |
|  | reverse | GAATCTCAGCTCTGAACACGTC |  |
| AC104758.2 | forward | CTCCCTGGGCCTGTTGTATCT | 60°C |
|  | reverse | TGTGCTGACCTTCAAGTTACG |  |
| AP000697.1 | forward | GGCCAGCACAAATAACAGGC | 60°C |
|  | reverse | TGACCTTCCCGAGAGCCA |  |
| LINC02460 | forward | CCAAGTGTCTCAAAGTGCCC | 60°C |
|  | reverse | CTCAGGGAGGTCCTGGAGAT |  |
| MAGEA8-AS1 | forward | GCTCCGTACTCTGCTGATGG | 60°C |
|  | reverse | GCTGTAGCTGCCAAAGTGAC |  |
| AC006150.1 | forward | TGGCTGTCACTATTCACTGTGG | 60°C |
|  | reverse | TTCTGATGAGGAGCCGCAAA |  |
| AL359502.1 | forward | TGGTCCTGCTGTCAAGTTCA | 60°C |
|  | reverse | TGGCTCCTCATGCAGTACAA |  |
| AC011595.1 | forward | TGTTCTCAGGGCACAGGATT | 60°C |
|  | reverse | AAACCGCCCACAGACTATCA |  |
| AP005229.2 | forward | GCTCCGTCATGCTCTTTCAG | 60°C |
|  | reverse | AGGGACAGCGATTTCTTGGA |  |
| LINC00024 | forward | CCTAGCGGTCCTGGAAAGGG | 60°C |
|  | reverse | CTGCTCCAGACAACACCCG |  |
| AC090377.1 | forward | ACACGCGAGAATCAGTGTCA | 60°C |
|  | reverse | ACCAAGAACATCGGCCTTTCC |  |
| AL137220.1 | forward | GCACTCTGTTGAAAATGGCAGT | 60°C |
|  | reverse | AGTGGGAATGCAAGAGGACG |  |
| AC009133.20 | forward | CTGAGTGCTCATGGGTTTCTAC | 60°C |
|  | reverse | CAAGGTGCCAGATTGGAAAATGA |  |
| AC023200.1 | forward | TTCTCTTCATTTGGGCTGCCA | 60°C |
|  | reverse | AGAAGAAAGATGGCGTGGGA |  |
| AL121894.2 | forward | TGTAAATGCCTCCCTTGGGC | 60°C |
|  | reverse | ACTCTTGCAGATGAAAGGTGC |  |
| AC107463.1 | forward | CAGGCAACCAAAATCCTCGTG | 60°C |
|  | reverse | CTTTTCTGCAGTCGTTTGGG |  |
| AC068888.2 | forward | GCTGCAGCAATAAAAGTGCTA | 60°C |
|  | reverse | CTGGGATTAGATGTGAGCCACT |  |
| miR-204-5p | RT-PCR | GTCGTATCCAGTGCAGGGTCCGAGGTATTCGCACTGGATACGACAGGCAT |  |
|  | forward | CGCGTTCCCTTTGTCATCCT | 60°C |
|  | reverse | AGTGCAGGGTCCGAGGTATT |  |
| U6 | RT-PCR | AACGCTTCACGAATTTGCGT |  |
|  | forward | CTCGCTTCGGCAGCACA | 60°C |
|  | reverse | AACGCTTCACGAATTTGCGT |  |
| BRD4 | forward | CTTTGAGACCCTGAAGCCGT | 60°C |
|  | reverse | TCGGAGCCATCTCTGTTTCG |  |
| GAPDH | forward | GGGGCTCTCCAGAACATCATCC | 60°C |
|  | reverse | ACGCCTGCTTCACCACCTCTT |  |
