# Supplementary figures and images for: LncRNA AL139294.1 can be transported by extracellular vesicles to promote the oncogenic behaviour of recipient cells through activation of the Wnt and NF-κB2 pathways in non-small-cell lung cancer
Source: J Exp Clin Cancer Res. 2024 Jan 16;43:20. doi: 10.1186/s13046-023-02939-z (PMC10790371; doi:10.1186/s13046-023-02939-z)

## Slide 1
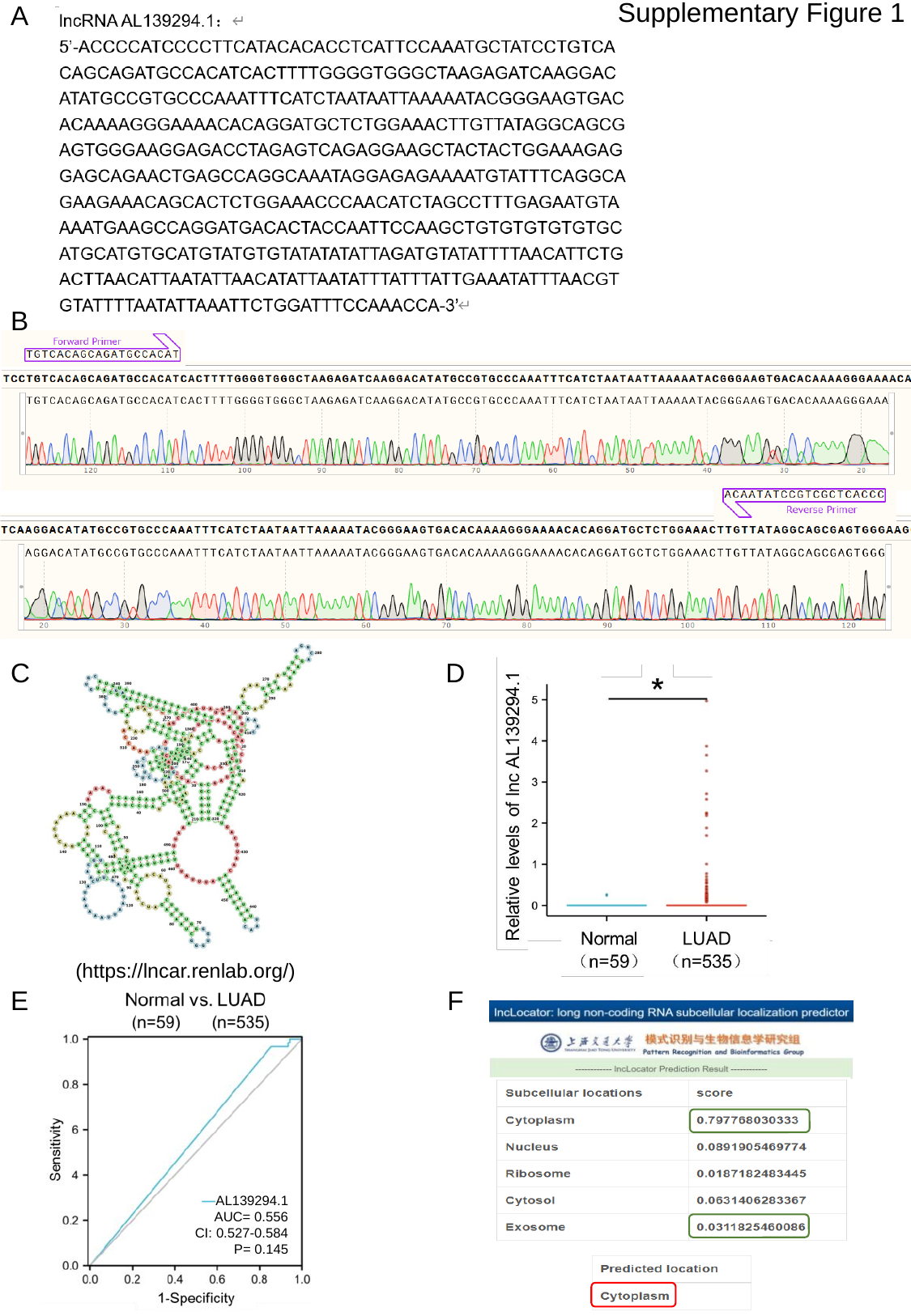

Supplementary Figure 1
A
B
(https://lncar.renlab.org/)
C
D
E
F
—AL139294.1
AUC= 0.556
CI: 0.527-0.584
P= 0.145

Supplement: Supplementary file 5 — Additional file 5: Fig. S1. The structure and cellular localization of AL139294.1. A The sequence of AL139294.1. B Sanger sequencing verified the specificity of the AL139294.1 amplification product. C The lnCAR database (https://lncar.renlab.org/) shows that AL139294.1 is 512 nt long, and its secondary structure contains multiple stem loops. D TCGA cohort (TCGA-LUAD dataset) shows the levels of AL139294.1 in normal (n = 59) and LUAD tissues (n = 535). E ROC analysis was performed to estimate the diagnostic efficacy of AL139294.1 in distinguishing normal (n = 59) and LUAD (n = 535). F The cellular location of AL139294.1 was checked by the lncLocator database (http://www.csbio.sjtu.edu.cn/bioinf/lncLocator/). *P < 0.05. [file 13046_2023_2939_MOESM5_ESM.pptx]

## Slide 1
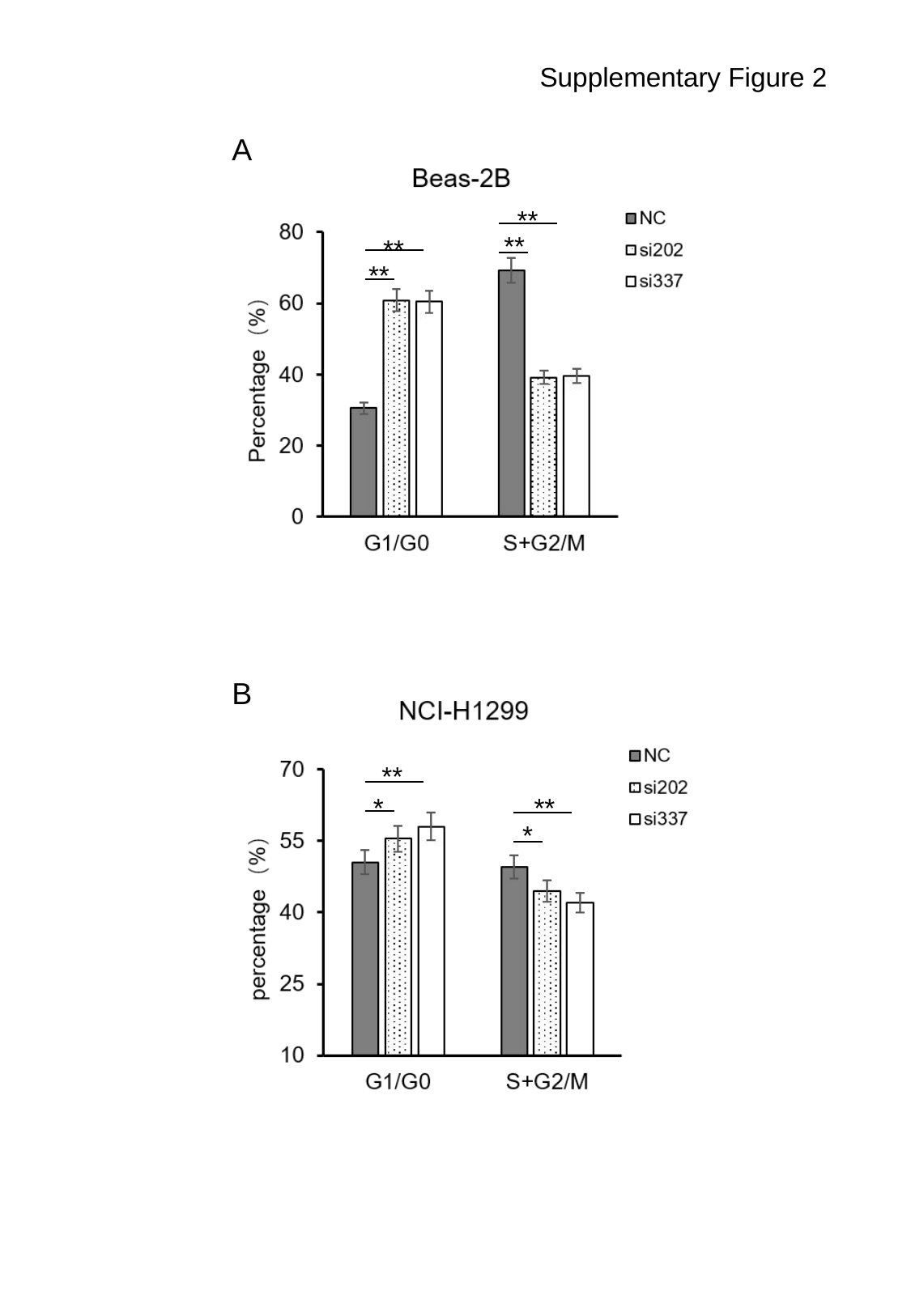

Supplementary Figure 2
A
**
**
**
**
B
**
**
*
*

Supplement: Supplementary file 6 — Additional file 6: Fig. S2. The effect of AL139294.1 knockdown on cells’ cycle. Flow cytometry detected the effect of AL139294.1 knockdown on Beas-2B (A) and NCI-H1299 (B) cell cycle. *P < 0.05 and **P < 0.01. [file 13046_2023_2939_MOESM6_ESM.pptx]

## Slide 1
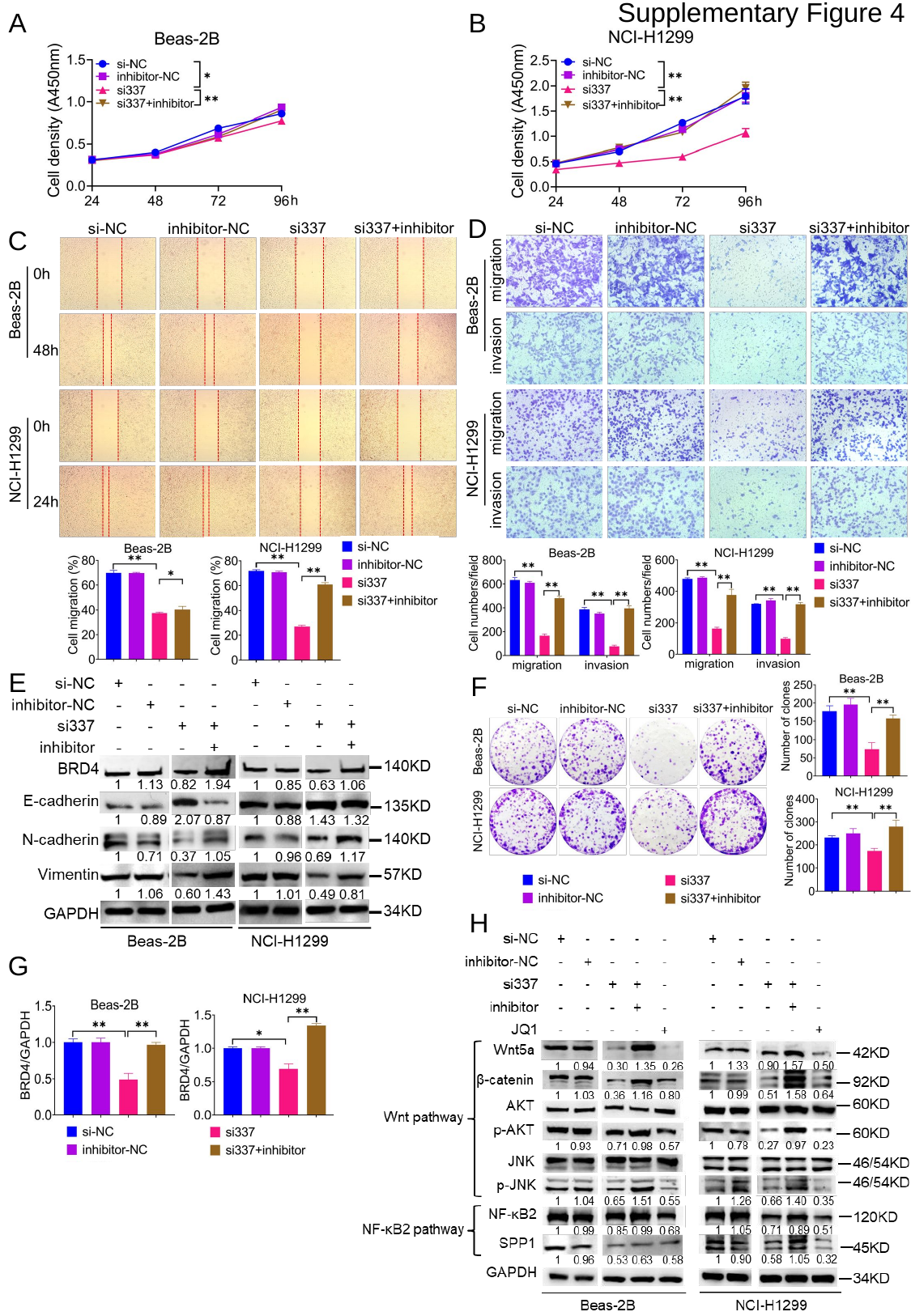

Supplementary Figure 4
A
B
D
C
E
F
H
G

Supplement: Supplementary file 8 — Additional file 8: Fig. S4. AL139294.1 promotes the tumorigenic capacities of the NSCLC cells by indirectly regulating BRD4 and activating the Wnt and NF-κB2 pathways. The effects of AL139294.1 knockdown, and the co-transfection of si337 and miR-204-5p inhibitors on the proliferation ability of Beas-2B (A) and NCI-H1299 (B) cells were examined by CCK-8 assay. Wound healing assay (C) and transwell assay (D) were used to evaluate the migration and invasion of Beas-2B and NCI-H1299 cells transfected with AL139294.1 si337 and miR-204-5p inhibitors. E Western blotting was carried out to test BRD4 and EMT-related proteins after the transfection of AL139294.1 si337 and miR-204-5p inhibitors. F Colony formation assay was performed to evaluate the colony formation ability of cells. G The mRNA levels of BRD4 in cells were detected by qPCR after the transfection of AL139294.1 si337 and miR-204-5p inhibitors. H Western blotting was used to detect the levels of Wnt5a pathway-related proteins (Wnt5a, β-catenin, AKT, and JNK) and NF-κB2 pathway-related proteins (NF-κB2, and SPP1) in Beas-2B and NCI-H1299 cells treated with AL139294.1 si337, miR-204-5p inhibitors or JQ1. *P < 0.05, **P < 0.01. [file 13046_2023_2939_MOESM8_ESM.pptx]

## Slide 1
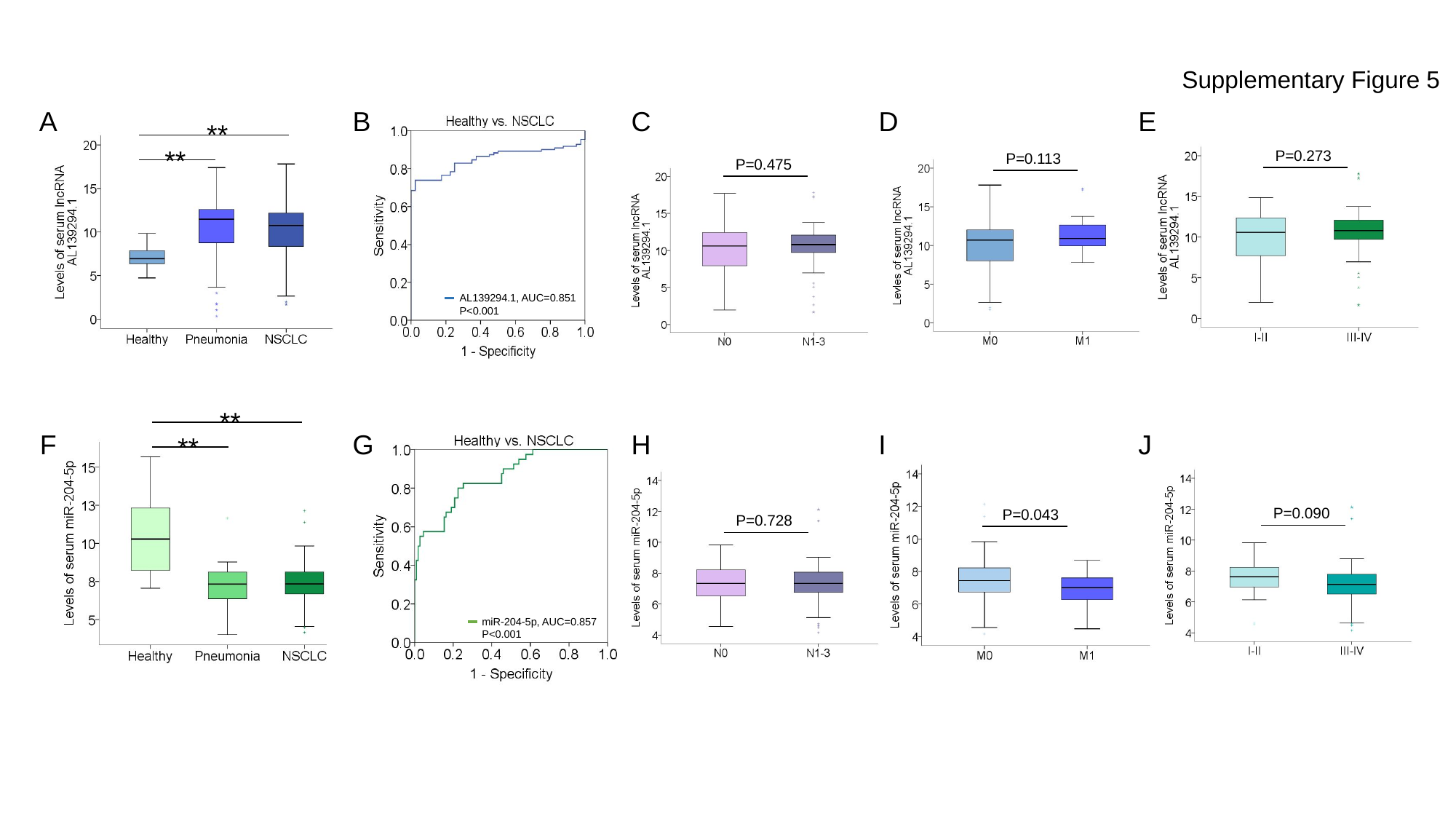

Supplementary Figure 5
A
B
C
D
E
AL139294.1, AUC=0.851
P<0.001
**
**
P=0.273
P=0.113
P=0.475
**
**
F
G
H
I
J
miR-204-5p, AUC=0.857
P<0.001
P=0.043
P=0.090
P=0.728

Supplement: Supplementary file 9 — Additional file 9: Fig. S5. The levels of serum AL139294.1 and miR-204-5p. The box plots show the relative levels of serum AL139294.1 (A) and miR-204-5p (F) in three cohorts: healthy (n = 40), pneumonia (n = 49), and NSCLC (n = 111). ROC analyses evaluated the diagnostic ability of serum AL139294.1 (B) and miR-204-5p (G) to differentiate healthy from NSCLC. The box plots show the levels of serum AL139294.1 (C, D, E) and miR-204-5p (H, I, J) in the subgroups of N0 (n=38) and N1-3 (n = 73), M0 (n = 88) and M1 (n = 23), and stages I-II (n=44) and III-IV (n = 67). **P < 0.01. [file 13046_2023_2939_MOESM9_ESM.pptx]
