# Supplementary material for: LncRNA AL139294.1 can be transported by extracellular vesicles to promote the oncogenic behaviour of recipient cells through activation of the Wnt and NF-κB2 pathways in non-small-cell lung cancer
Source: J Exp Clin Cancer Res. 2024 Jan 16;43:20. doi: 10.1186/s13046-023-02939-z (PMC10790371; doi:10.1186/s13046-023-02939-z)
Supplement: Supplementary file 7 — Additional file 7: Fig. S3. Prediction of AL139294.1–miR-204-5p–BRD4 regulatory axis. A The miRDB database (http://mirdb.org) was used to predict miRNAs that interact with AL139294.1. B MiR-204-5p was found to have the binding site with AL139294.1. C miRWalk database (http://mirwalk.umm.uni-heidelberg.de) was used to predict the binding sites of miR-204-5p and BRD4. Survival analyses (Kaplan-Meier plotter (kmplot.com)) show the association of BRD4 mRNA levels with first-progression (D, n = 874), and post-progression survival (E, n = 242) of patients with lung cancer. The cutoff is an auto-cutoff at which a significant difference is obtained between the lower and higher expression levels. F, G CPTAC cohort (LUAD dataset) analyses present higher protein levels of BRD4 in LUAD tissues (n = 109) than those in normal tissues (n = 102) as well as paired tissues (normal, n = 102; LUAD, n = 102). H The protein levels of BRD4 are different in normal tissues (n = 11) and LUAD tissues with stages 1 (n = 59), 2 (n = 30), 3 (n = 21), and 4 (n = 1) based on the CPTAC cohort (LUAD dataset). The data were transformed by Log 2. **P < 0.01. [file 13046_2023_2939_MOESM7_ESM.pptx]

## Slide 1
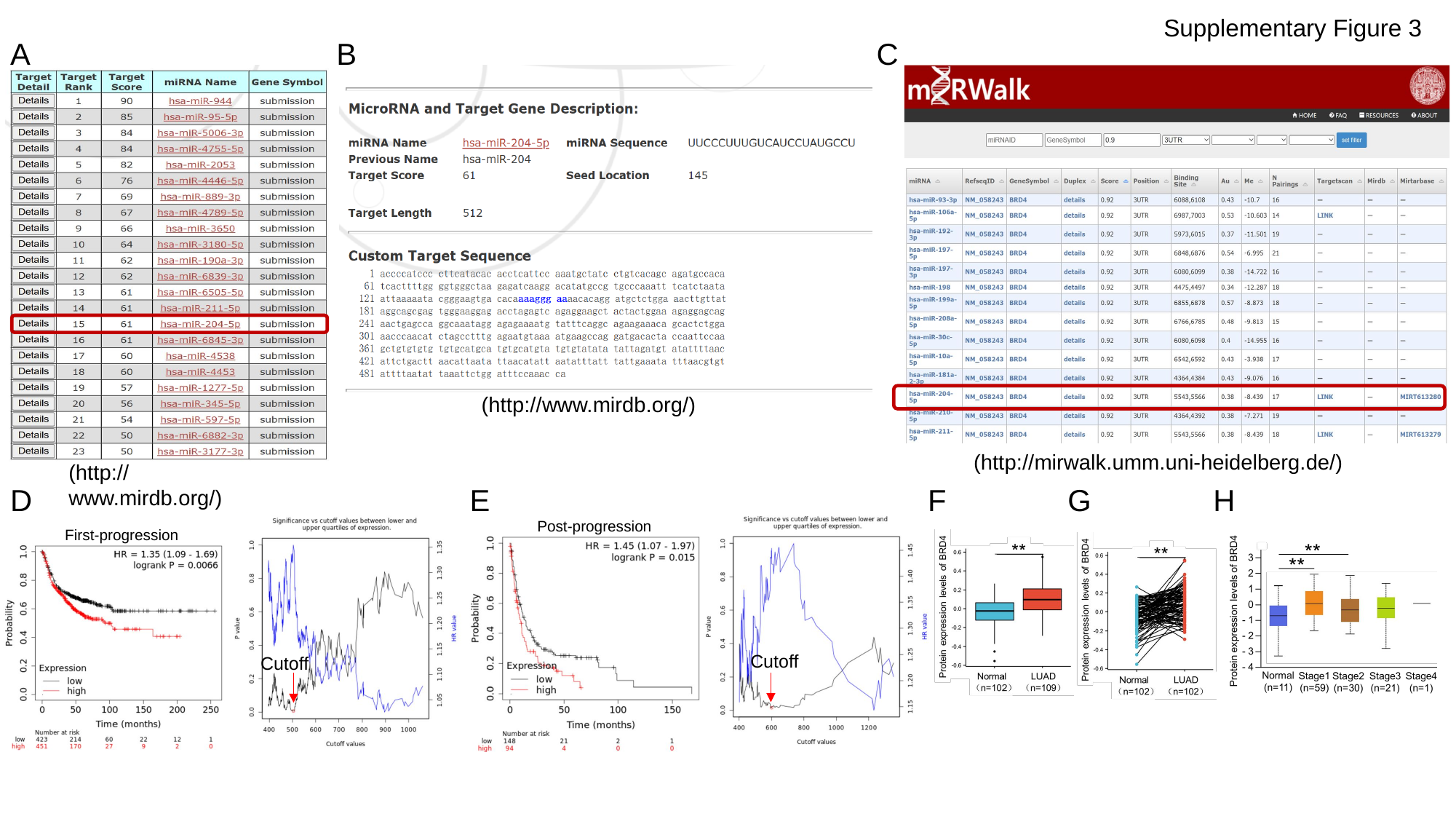

Supplementary Figure 3
A
B
C
(http://www.mirdb.org/)
(http://www.mirdb.org/)
(http://mirwalk.umm.uni-heidelberg.de/)
D
E
F
G
H
Post-progression
Cutoff
Cutoff
First-progression
